# Transcriptomic Analysis of Thermally Stressed *Symbiodinium* Reveals Differential Expression of Stress and Metabolism Genes

**DOI:** 10.3389/fpls.2017.00271

**Published:** 2017-02-28

**Authors:** Sarah L. Gierz, Sylvain Forêt, William Leggat

**Affiliations:** ^1^College of Public Health, Medical and Veterinary Sciences, James Cook University, TownsvilleQLD, Australia; ^2^Comparative Genomics Centre, James Cook University, TownsvilleQLD, Australia; ^3^ARC Centre of Excellence for Coral Reef Studies, James Cook University, TownsvilleQLD, Australia; ^4^Evolution, Ecology and Genetics, Research School of Biology, Australian National University, CanberraACT, Australia

**Keywords:** *Symbiodinium*, dinoflagellates, transcriptome, RNA-Seq, gene expression, thermal stress

## Abstract

Endosymbioses between dinoflagellate algae (*Symbiodinium* sp.) and scleractinian coral species form the foundation of coral reef ecosystems. The coral symbiosis is highly susceptible to elevated temperatures, resulting in coral bleaching, where the algal symbiont is released from host cells. This experiment aimed to determine the transcriptional changes in cultured *Symbiodinium*, to better understand the response of cellular mechanisms under future temperature conditions. Cultures were exposed to elevated temperatures (average 31°C) or control conditions (24.5°C) for a period of 28 days. Whole transcriptome sequencing of *Symbiodinium* cells on days 4, 19, and 28 were used to identify differentially expressed genes under thermal stress. A large number of genes representing 37.01% of the transcriptome (∼23,654 unique genes, FDR < 0.05) with differential expression were detected at no less than one of the time points. Consistent with previous studies of *Symbiodinium* gene expression, fold changes across the transcriptome were low, with 92.49% differentially expressed genes at ≤2-fold change. The transcriptional response included differential expression of genes encoding stress response components such as the antioxidant network and molecular chaperones, cellular components such as core photosynthesis machinery, integral light-harvesting protein complexes and enzymes such as fatty acid desaturases. Differential expression of genes encoding glyoxylate cycle enzymes were also found, representing the first report of this in *Symbiodinium*. As photosynthate transfer from *Symbiodinium* to coral hosts provides up to 90% of a coral’s daily energy requirements, the implications of altered metabolic processes from exposure to thermal stress found in this study on coral-*Symbiodinium* associations are unknown and should be considered when assessing the stability of the symbiotic relationship under future climate conditions.

## Introduction

Unicellular dinoflagellates (genus *Symbiodinium*) form symbiotic relationships with reef-building corals and other marine invertebrates. The success of coral reef ecosystems is due to mutualistic nutrient exchange between host and endosymbionts (Yellowlees et al., 2008). Exposure to stressors (e.g., elevated temperature) has been attributed as causing coral bleaching, the dysfunction of the symbiotic relationship, resulting in the expulsion of *Symbiodinium* from the coral host. Coral bleaching is the release of either the *Symbiodinium* cells from host tissue or the loss of their photosynthetic pigments (Iglesias-Prieto et al., 1992). Depending on the degree of the bleaching event the result may vary, with the host being recolonised by *Symbiodinium*, disease outbreak or widespread coral mortality (Hoegh-Guldberg, 1999).

Experimentation on *Symbiodinium* and the coral holobiont has focused on many environmental factors implicated in the onset of coral bleaching including elevated seawater temperatures, acidification, eutrophication (nutrient stress), and disease. The effect of high sea-surface temperatures have been a key focus due to mass coral bleaching events [∼42% GBR reefs bleached in 1998 and ∼54% reefs bleached in 2002 (Berkelmans et al., 2004)], attributed to global climate change (Hoegh-Guldberg, 1999) with the 1998 bleaching event coinciding with an El Niño Southern Oscillation event (Bruno et al., 2001; Fujise et al., 2014). Modeling of bleaching patterns have shown that short periods of high temperature are highly stressful to corals (Berkelmans et al., 2004) with studies emulating these acute conditions in an attempt to understand mechanisms of coral bleaching (Iglesias-Prieto et al., 1992; Ralph et al., 2001, 2005; Takahashi et al., 2008). These studies though extremely valuable in providing an insight into bleaching processes, employ experimental conditions that are not reflective of future long-term predicted sea-surface temperatures or coral bleaching induced by moderate thermal stress over long periods of time (Berkelmans et al., 2004; Fujise et al., 2014; Ainsworth et al., 2016). Additionally mechanisms of thermal acclimation within *Symbiodinium* are also unknown, with only two recent studies investigating the effect of moderate thermal stress on photobleaching (Takahashi et al., 2013; Fujise et al., 2014).

Dinoflagellates have a number of cellular traits and features that make them unique. Dinoflagellates have large nuclear genomes, the chromosomes remain permanently condensed through the cell cycle and contain highly expressed genes with elevated copy numbers and tandem repeats (Hackett et al., 2004). Approximately half of the dinoflagellates are photosynthetic, having acquired a variety of plastids via endosymbiotic events (Delwiche, 1999; Hackett et al., 2004). In general, the plastids are surrounded by three envelope membranes and have unique chloroplast genome structure, having been reduced to single gene minicircles, with the majority of genes transferred to the nucleus (Hackett et al., 2004; Barbrook et al., 2014; Mungpakdee et al., 2014).

The genus *Symbiodinium* is a taxonomically diverse species complex, divided into nine phylogenetically distinct clades (A-I; Pochon and Gates, 2010). Additionally intra-cladal diversity exists subdividing the genus further. Associations between *Symbiodinium* exist with many taxa including ciliates, platyhelminthes, and a variety of marine invertebrates such as cnidarians, molluscs, poriferans, and foraminiferans (Baker, 2003; Coffroth and Santos, 2005; Stat et al., 2008; Venn et al., 2008; Pochon et al., 2010). *Symbiodinium* associations may either exist as mixed populations, at intra-clade or inter-clade level, or as host–symbiont specific interactions (Baker, 2003; Coffroth and Santos, 2005; Pochon and Gates, 2010). Mixed *Symbiodinium* populations may occur in different proportions, with a single dominant species with multiple species detected at low abundances (Baker, 2003). Therefore, understanding the different *Symbiodinium* strains may provide an insight into the complexity of symbiotic interactions that are observed. Development of high throughput sequencing technologies has seen many advances in *Symbiodinium* genomics and transcriptomics [summarized in Shinzato et al. (2014)]. These include publication of three *Symbiodinium* nuclear genomes, *Symbiodinium minutum* (clade B1; Shoguchi et al., 2013), the *Symbiodinium kawagutii* (clade F1) nuclear genome (Lin et al., 2015), and the *Symbiodinium microadriaticum* (clade A1) nuclear genome (Aranda et al., 2016) and a further 13 sequencing projects representing organelle genomes and transcriptomes of various *Symbiodinium* sp. (clades A–D and F; Suppleentary Table S1). The *Symbiodinium minutum* (clade B) genome has identified a large number of protein-coding genes (41,925), attributed to duplication events due to the presence of highly repetitive gene clusters (Shoguchi et al., 2013; Shinzato et al., 2014). Further, expansions of regulator of chromosome condensation family protein (RCC1) provide a possible molecular basis for permanently condensed chromatin observed in dinoflagellates (Shoguchi et al., 2013; Shinzato et al., 2014). Other expanded multi-copy gene families identified include ion channel proteins and the chlorophyll *a*/*b*-binding proteins (*lhcb*, PF00504; Shoguchi et al., 2013). Study of the *S. minutum* mitochondrial genome (Shoguchi et al., 2015) has shown that it is greatly expanded and fragmented, whereas, the plastid genome is greatly reduced with most (all but 14 located on DNA minicircles) plastid-associated genes being transferred to the nucleus (Mungpakdee et al., 2014). Mechanisms for RNA editing in *Symbiodinium* have also been revealed from studying the plastid genome (Mungpakdee et al., 2014). *Symbiodinium* transcriptomes and EST datasets have been published using different sequencing technologies representing various clades that associate with a variety of hosts (summarized in Leggat et al., 2011b; Rosic et al., 2014; Shinzato et al., 2014; Xiang et al., 2015; Levin et al., 2016). Due to the interest in studying establishment of symbiosis, gene expression studies have focused on genes associated with these molecular mechanisms (Voolstra et al., 2009a; Mohamed et al., 2016).

*Symbiodinium* genome and transcriptome data have allowed for studies of specific gene families of interest (Shinzato et al., 2014). Integral light-harvesting complex (LHC) gene families [chlorophyll *a*-chlorophyll *c_2_*-peridinin protein complex (acpPC)] in *Symbiodinium* have been investigated due to their unique gene arrangement (annotated as chlorophyll *a*/*b*-binding proteins in some instances; Takahashi et al., 2008; Boldt et al., 2012; Maruyama et al., 2015; Xiang et al., 2015). Gene-mining has revealed high diversification of the integral light-harvesting gene family (*acpPC*) in *Symbiodinium* species (Boldt et al., 2012; Maruyama et al., 2015), supporting theories of intra- and intergenic duplication from ancestral LHC gene(s) possibly of red algal origin (Engelken et al., 2010; Boldt et al., 2012; Maruyama et al., 2015). The functional purpose for the highly redundant multicopy gene family has been hypothesized as photoprotective (Boldt et al., 2012; Maruyama et al., 2015; Xiang et al., 2015), and differential expression of *acpPC* genes in *Symbiodinium* has been detected under thermal stress in targeted gene expression studies (Takahashi et al., 2008; Gierz et al., 2016). This study investigates the expression of *Symbiodinium* sp. (clade F) light-harvesting *acpPC* genes under thermal stress at the transcriptome level.

Whilst these next-generation sequencing projects have generated large quantities of data and provide basal reference transcriptomes, some of these studies lack comparison between stress and non-stress conditions, which is one of the aims of this study. Recently, the transcriptional response of cultured *Symbiodinium* (strain SSB01) maintained under different light intensities with different growth conditions (Xiang et al., 2015), and of two type C1 *Symbiodinium* cultures exposed to a 13-days thermal stress (32°C) (Levin et al., 2016) have been published, and are providing insights into *Symbiodinium* transcriptomes under stress conditions. Previous studies of *Symbiodinium* using targeted gene expression analysis (quantitative-PCR), demonstrate that alteration of gene expression generally occurs at low fold changes, with regulation hypothesized to occur instead through translational or post-translational mechanisms (Leggat et al., 2011a; Rosic et al., 2011; Krueger et al., 2015; Gierz et al., 2016). This study employed Illumina RNA-Seq and used four biological replicates at each time point per condition to ensure a robust analysis. Samples were selected for sequencing to encapsulate stages of exposure to elevated temperatures at beginning (day 4), middle (day 19), and end (day 28) of the experimental period. This experimental design has allowed us to investigate the potential effects of exposure to elevated temperatures on *Symbiodinium* molecular processes.

## Materials and Methods

### Culture Conditions and Experimental Design

Cultures of *Symbiodinium* sp. [clade F (ITS2 rDNA region; Supplementary Table S2)] were obtained from the Australian Institute of Marine Science (AIMS). Cells were grown in ASP-8A media (Blank, 1987), in 75 cm^2^ vented tissue culture flasks at 25°C. Cultures were maintained in light cabinets (Thermoline Scientific refrigerated incubator, Sanyo) at an irradiance of 80–100 μmol quanta m^-2^ s^-1^ measured using a LI-193SA Underwater Spherical Quantum Sensor with a LI-250A Light Meter (LI-COR^®^ Inc., Lincoln, NE, USA).

**FIGURE 1:**
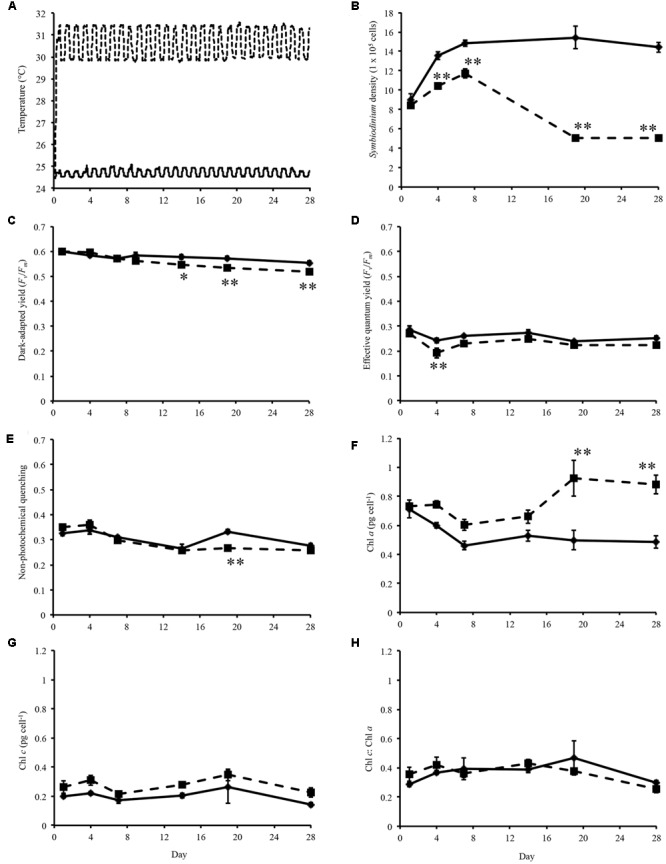
**Experimental temperatures and physiological data. (A)** Temperature of control (solid line) and heated treatment (dashed line) during the 28 days thermal experiment. **(B)** *Symbiodinium* cell density throughout experiment. **(C)** Dark-adapted yield (*F*_v_*/F*_m_) of *Symbiodinium* cells during the experiment. **(D)** Effective quantum yield of *Symbiodinium* cells at the end of the induction phase. **(E)** Non-photochemical quenching of *Symbiodinium* cells at the first data point of the recovery phase. **(F)** Concentration of chlorophyll *a* in *Symbiodinium* cells. **(G)** Concentration of chlorophyll *c* in *Symbiodinium* cells. **(H)** Ratio of chlorophyll *a* to chlorophyll *c* per *Symbiodinium* cell. *Symbiodinium* cells exposed to control temperatures (solid line) and thermal treatment (dashed line). *Error bars* represent ± SEM, *n* = 5, some *error bars* obscured by data point markers. The statistical difference (*post hoc* sequential Bonferroni analysis) between treatment and control is indicated as ^∗^*p* < 0.05 or ^∗∗^*p* < 0.01.

Over a period of 28 days cultures of *Symbiodinium* were exposed to control temperatures (24.5–25°C) or thermal treatment temperatures of approximately (30–31.5°C; **Figure 1A**). In both treatment and controls daily fluctuations in temperature reflect 12:12 h light:dark photoperiod. Temperatures in each incubator were recorded every 10 min with HOBO^®^ temperature/alarm pendant data loggers (Onset, MA, USA). To maintain an adequate number of cells throughout the experiment 16 flasks were designated to each treatment with five replicate culture flasks sampled at each time point per treatment (Supplementary Figure S1). Samples were taken at 10 h into the light photoperiod.

### *Symbiodinium* Density and Chlorophyll Pigment Analysis

On days 1, 4, 7, 19, and 28, five replicate culture flasks were taken from each treatment for cell number approximation and pigment quantification. Cell numbers were determined using a Neubauer haemocytometer, with replicate cell counts performed (*n* = 4). *Symbiodinium* cells were pelleted by centrifugation at 4500 × *g* for 3 min for chlorophyll *a* and *c* quantification. Chlorophylls were extracted in 90% acetone for 20 h in the dark at 4°C and pigment content quantified using the equations of Jeffrey and Humphrey (1975).

### Imaging-Pulse Amplitude–Modulated Fluorometry

Imaging-Pulse amplitude-modulated (PAM) fluorometry (MAXI Imaging-PAM and ImagingWin software, Walz, Effeltrich, Germany) were used to measure photosynthetic efficiency of *Symbiodinium* cultures. The preprogrammed Induction Curve + Recovery kinetic recording type were used to examine the ability of *Symbiodinium* to dissipate excess light energy and recover from light stress after exposure to elevated temperature. *Symbiodinium* cells were aseptically transferred to 15 mL falcon tubes and pelleted by centrifugation at 4,500 × *g* for 3 min. Cell pellets were resuspended in approximately 250 microliters ASP-8A media and transferred to a 96-well plate and dark-adapted for 20 min prior to analysis. Following dark-adaption, minimum chlorophyll fluorescence (*F*_o_) were determined using blue measuring light (intensity 2), and maximum chlorophyll fluorescence (*F*_m_) were determined by applying a pulse (0.72 s) of saturating light (intensity 5, ∼2800 μmol quanta m^-2^ s^-1^) allowing calculation of the dark-adapted maximal quantum yield of PS II (*F*_v_*/F*_m_). For the Induction Curve, actinic illumination (254 μmol quanta m^-2^ s^-1^, intensity 6) was switched on and 15 saturating pulses of photosynthetically active radiation [∼2800 μmol quanta m^-2^ s^-1^ (intensity 5, 0.72 s)] were applied at 20 s intervals for 5 min. During the Recovery phase, a further 16 saturation pulses were applied within a 14 min period without actinic illumination, time between each pulse exponentially increased. Imaging-PAM fluorometry were used to determine photo-kinetic parameters, such as the maximal quantum yield of PS II (*F*_v_*/F*_m_), effective quantum yield at the end of the induction curve, and non-photochemical quenching (NPQ) at the beginning of the recovery phase. Light levels were measured using a LI-190SA Quantum Sensor with a LI-250A Light Meter (LI-COR^®^ Inc., Lincoln, NE, USA).

### Data Analysis

Statistics software package SPSS statistics (v19.0, IBM, USA) were used for all statistical analyses of physiological parameters. A generalized linear model with ‘day’ and ‘treatment’ as main effects and ‘day × treatment’ as an interaction were used for pairwise comparison of cell density, chlorophyll *a* and *c*, and imaging-PAM with sequential Bonferroni *post hoc* test to determine significant differences between control and treatment. The generalized linear model approach was chosen as samples taken at each time point were considered independent.

### RNA Isolation and Sequencing

For total RNA isolation, 15 mL of cells were pelleted by centrifugation at 4,500 × *g* for 3 min. Cells were then transferred to a screw cape tube and centrifuged at 8,050 × *g* for 3 min. Pellets were snap frozen in liquid nitrogen and stored at -80°C. Total RNA were extracted using the RNeasy Plant Mini Kit (Qiagen, USA). *Symbiodinium* cells were first lysed using the FastPrep^®^-24 sample preparation system (MP Biomedicals, Australia). Four hundred and fifty microliters of Buffer RLT containing 1% β-mercaptoethanol were used to resuspend cells and were transferred to a lysing matrix D tube (MP Biomedicals, Australia), and shaken three times for 40 s at 4.5 M s^-1^ to lyse the cells. Total RNAs were isolated from cells using the Purification of Total RNA from plant cells and tissues protocol. The optional on-column DNase Digestion was performed using the RNase-Free DNase set (Qiagen, USA) as per the manufacturer’s protocol.

Concentrations of isolated total RNAs were checked using a NanoDrop ND-1000 spectrophotometer (NanoDrop Technologies, Wilmington, DE, USA) and quality were assessed using a Bioanalyzer (Agilent) prior to library generation. RNA-Seq libraries were prepared by the Australian Genome Research Facility (AGRF, Melbourne, Australia), using the Illumina TruSeq RNA sample preparation kit V2 (Illumina) and the standard Illumina protocols. Multiplexed sequencing were performed on the 24 libraries by AGRF on an Illumina HiSeq 2000 platform on two lanes, generating over 370 million 100 bp single-end reads.

### RNA-Seq Analysis

Image analyses were performed in real time by the HiSeq Control Software (HCS) v1.4.8 and Real Time Analysis (RTA) v1.18.61, running on the instrument computer. The Illumina CASAVA (Consensus Assessment of Sequence and Variation) 1.8.2 pipeline was used to generate the sequence data. Sequencing reads were filtered according to their multiplexing tags and multiplexing tags were removed. The sequenced library were mapped against a *Symbiodinium* reference transcriptome generated from the same culture (Bobeszko et al., unpublished). Briefly, for the *Symbiodinium* reference transcriptome (BioProject number PRJNA371519), paired-end reads were trimmed, removing sequence adaptors and low-quality regions using libngs^1^ with a minimum quality of 20 bp and a minimum size of 75 bp. Trimmed reads were then assembled with Trinity (Grabherr et al., 2011) and the resulting assembly clustered using CD-HIT-EST (Fu et al., 2012) using 90% similarity and a word size of 8. TransDecoder (Haas et al., 2013) and Blast2GO (Conesa et al., 2005) were used to predict protein-coding sequences.

**FIGURE 2:**
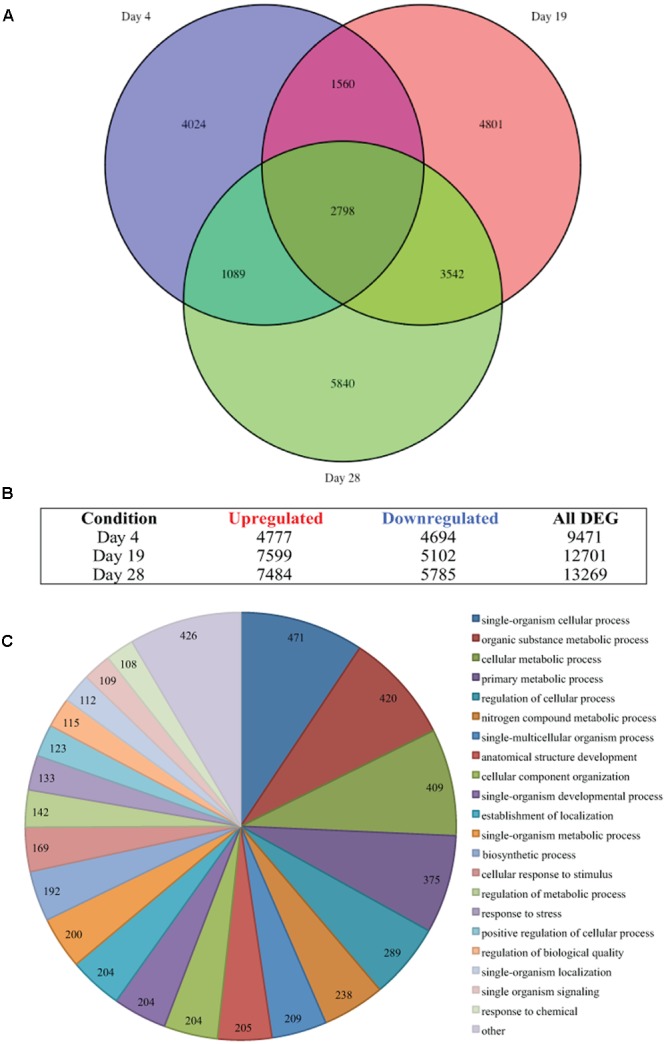
**Thermal stress-induced differential gene expression. (A)** Venn diagram illustrates the differential expression of 35,441 genes (FDR < 0.05) of *Symbiodinium* sp., after exposure to thermal stress for 4, 19, and 28 days. Venn diagram generated using the VennDiagram package in R **(B)** Illustration of the distribution of upregulated or downregulated genes (FDR < 0.05) at days sampled following exposure to thermal stress. **(C)** Visualization of the distribution of biological process gene ontology (GO) classifications for the 2,798 genes differentially expressed at all time points in *Symbiodinium* exposed to thermal stress (FDR < 0.05). GO annotation graph produced using Blast2GO, GO categories displayed at ontology level 3 and slices smaller than 2% grouped into the ‘other’ term, numbers displayed represent the number of sequences assigned to each ontology category.

The single-end sequenced library were mapped using the ArrayStar application and QSeq module of the DNAStar Lasergene Genomics Suite (Version 11; DNASTAR, Inc., Madison, WI, USA) with all parameters set to defaults. QSeq parameters were set to default, read counts were normalized via RPKM (reads per kilo base of exon model per million mapped reads; Mortazavi et al., 2008) and processed genes defined as ‘use sequences as genes.’ The RPKM method standardizes molar concentration of transcripts by determining transcript length (in kilobases) and the read abundances by dividing each read count by the library-size (in millions) to normalize (Mortazavi et al., 2008). The RPKM normalization method is accepted in RNA-Seq analysis as it removes technical biases introduced by sequence-length and library-size (Li et al., 2015; Conesa et al., 2016), and is suitable for the single-end reads generated from sequencing. Statistically significant expression changes between the control and treatment data sets were determined using the student’s *t*-test with multiple test correction by Benjamini–Hochberg false discovery rate (FDR < 0.05). The results for each time point comparison [day 4, 9,471 DEG; day 19, 12,701 DEG; and day 28, 13,269 DEG (**Figures 2A,B**)], uniquely differentially expressed at any time point (23,654 DEG) and differentially expressed at every time point (2,798 DEG) were exported and saved in Microsoft Excel (Microsoft, Redmond, WA, USA) and used for subsequent analysis.

Nucleotide fasta files for candidate transcripts were generated using the ‘SeqinR’ package^2^ (Charif and Lobry, 2007), venn diagrams were drawn with the ‘VennDiagram’ package^3^ (Chen and Boutros, 2011), using R version 3.3.2^4^ (R Core Team, 2014). Distribution of gene ontology (GO) terms within the sequences that were differentially expressed at all time point (2,798 DEG) were determined using Blast2GO (v3.3.5; Conesa et al., 2005). Briefly, nucleotide sequences were imported into Blast2GO and GO terms generated using default parameters for the blast (blastx), mapping and annotation steps (Conesa and Götz, 2008). To visualize GO term distributions, combined GO annotation graphs were produced (default settings) and used to generate graph level pie charts of biological processes (**Figure 2C**), molecular function (Supplementary Figure S2), and cellular component (Supplementary Figure S3), sequences were filtered by GO terms and a cut-off ontology level of 3 were applied, slices smaller than 2% were grouped in the “other” slice. To further analyze the distribution of GO terms within the biological process category data were split into three sets, significantly upregulated at all time points (1,428 DEG), significantly downregulated at all time points (1,331 DEG), or significantly expressed (up and down) at all time points (39 DEG; Supplementary Figure S4). Sequences with GO terms contributing to biological process categories were selected/filtered and used to further identify genes and pathways differentially expressed over all time points. Heatmaps were drawn with the ‘pheatmap’ package^5^ (Kolde, 2015).

### Data Deposition

The Illumina sequenced read data reported in this article have been deposited into the National Center for Biotechnology Information (NCBI) Sequence Read Archive under the accession number SRA467551, which is associated with BioProject number PRJNA342240.

## Results

### Physiological Responses of *Symbiodinium* to Thermal Stress

*Symbiodinium* cell densities were significantly decreased in thermally stressed cultures from day 4 onward of the experiment (**Figure 1B**). Flasks sampled on day 1 were sampled again on day 14, day 4 again on day 19, and day 7 again on day 28.

Maximum quantum yield of photosynthesis, *F*_v_*/F*_m_, were measured on sampling days throughout the experiment. For cultures maintained at control temperatures dark-adapted yield ranged between 0.536 and 0.616 (average 0.577, standard error ± 0.003; **Figure 1C**). Analysis of the dark-adapted yield found that there were a significant interaction between treatment and day (*p* < 0.001, *df* = 5; **Figure 1C**) and for days (*p* < 0.001, *df* = 5) and treatments (*p* < 0.001, *df* = 1). Sequential Bonferroni *post hoc* analysis found that *F*_v_*/F*_m_ decreased in the heated treatment on day 14 (*p* < 0.05), day 19 (*p* < 0.01), and day 28 (*p* < 0.01) of the experiment (**Figure 1C**). *Symbiodinium* effective quantum yield at the end of the induction phase were also determined (**Figure 1D**). This measurement is taken after the cells were exposed to a phase of actinic light with periodic saturating pulses. Cells maintained at ∼31°C only exhibited decreased effective quantum yield on day 4 (*p* < 0.01; **Figure 1D**). NPQ a proxy for cell protective mechanisms was measured over the course of the experiment. Analysis of NPQ found that there were significant effects in the interaction between day and treatment (*p* < 0.001, *df* = 5) and between days (*p* < 0.001, *df* = 5; **Figure 1E**). NPQ values did vary slightly from controls in thermally stressed cells, though it only significantly decreased on day 19 (*p* < 0.001; **Figure 1E**).

Chlorophyll *a* content per *Symbiodinium* cell were increased in treatment cells compared to control cells over the experimental period (**Figure 1F**). Analysis of chlorophyll *a* content found that there were significant interaction day × treatment (*p* < 0.001, *df* = 5; **Figure 1F**) and differences between day (*p* < 0.001, *df* = 5) and treatment (*p* < 0.001, *df* = 1). Though chlorophyll *c* content were increased in thermally stressed cells no significant differences were found between control and treatment cells (**Figure 1G**). No significant differences were found in the ratio of chlorophyll *c* to chlorophyll *a* in *Symbiodinium* cells throughout the experiment (**Figure 1H**).

### Differential Gene Expression at a Pre-Bleaching Temperature Threshold

Differential gene expression in *Symbiodinium* in response to elevated temperature were determined at days 4, 19, and 28. Overall, the number of unique DEGs detected throughout the thermal stress accounts for 37.01% of the transcriptome (FDR < 0.05). Though a large number of differentially regulated genes were detected, only 2.78% (1,776 contigs) were unique genes with ≥2-fold change in expression. Comparison of gene expression between all time points identified 2,798 common transcripts differentially expressed (both up and down) under elevated temperature at all time points (**Figure 2A**) and these were further analyzed. Gene OntologyGO analysis of these common transcripts identified biological processes including 409 genes encoding proteins for cellular metabolic processes, 204 genes encoding proteins involved in cellular component organization, and 133 genes encoding proteins associated with response to stress (**Figure 2C**).

### Stress Response

**FIGURE 3:**
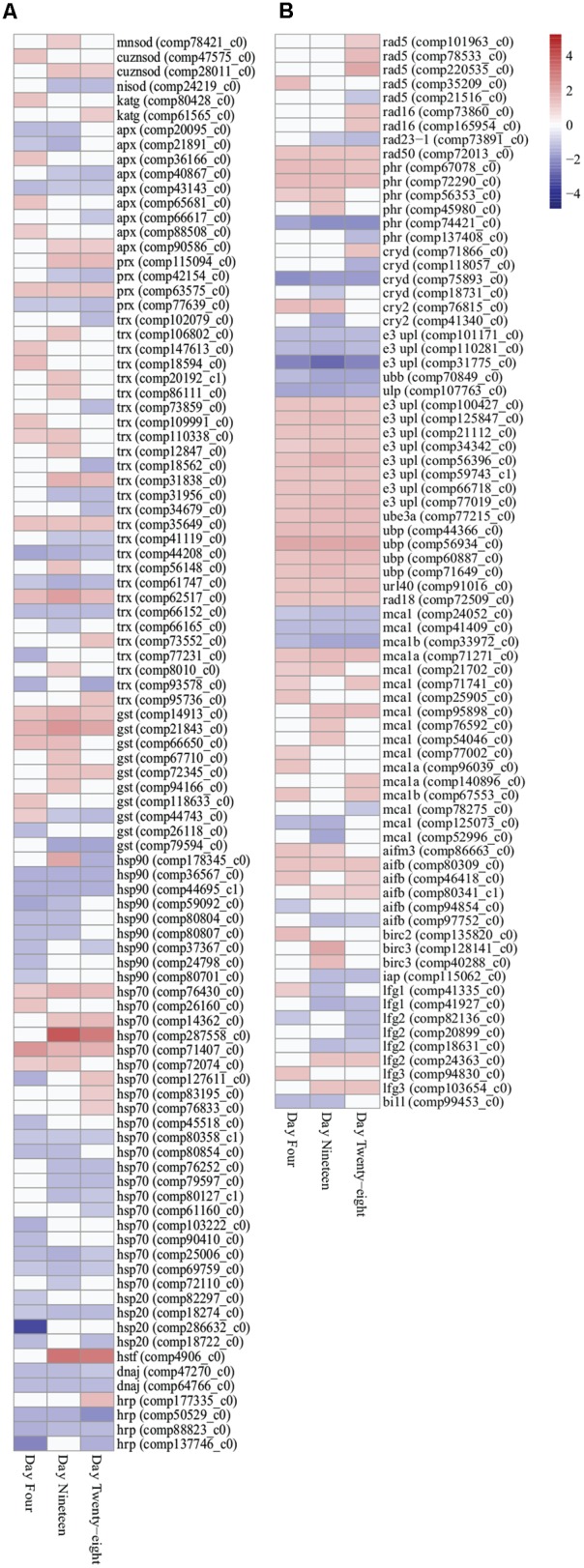
**Heatmap illustration of differentially expressed stress response genes (FDR < 0.05) in *Symbiodinium* exposed to thermal stress at days 4, 19, and 28.** Data are expressed as fold-changes relative to control; only significant data are shown (*p* < 0.05), non-significant data denoted as white boxes. **(A)** Differential expression of antioxidant defenses (enzymatic and non-enzymatic antioxidants) and molecular chaperones. **(B)** Differential expression of stress related transcripts including genes encoding DNA damage repair proteins, selected ubiquitin proteasome pathway components, metacaspases, and anti-apoptosis proteins. Abbreviations: cuznsod, copper-zinc superoxide dismutase; mnsod, manganese superoxide dismutase; nisod, nickel superoxide dismutase; katg, catalase peroxidase; apx, ascorbate peroxidase; prx, peroxiredoxin; trx, thioredoxin; gst, glutathione *S*-transferase; hsp90, heat shock protein 90; hsp70, heat shock protein 70; hsp20, heat shock protein 20; hrp, heat shock-related protein; dnaj, chaperone DNAJ; hstf, heat stress transcription factor; phr, DNA photolyase; cryd, cryptochrome DASH; cry2, cryptochrome 2; e3 upl, e3 ubiquitin-protein ligase; ube, ubiquitin-protein ligase 3a; ubp, ubiquitin carboxyl-terminal hydrolase; url40, ubiquitin ribosomal protein l40; ubb, polyubiquitin-b; ulp, ubiquitin-like specific protease; mca, metacaspase; aif, apoptosis-inducing factor; bir, baculoviral IAP repeat-containing protein; iap, inhibitor of apoptosis; lfg, protein lifeguard; bi1l, Bax inhibitor-like protein. Heatmaps generated using the ‘pheatmap’ package.

Antioxidant genes important in stress responses were detected with differential expression in thermally stressed cells. Superoxide dismutase (SOD) transcripts displayed increased expression throughout the experiment. Manganese SOD (MnSOD) expression were significantly increased at day 19 (comp78421_c0, 1.166 up, *p* < 0.03), whereas, copper/zinc SOD (CuZnSOD) were significantly upregulated at day 4 (comp47575_c0, 1.243 up, *p* < 0.03) and for transcript comp28011_c0 at day 19 (1.187 up, *p* < 0.02) and day 28 (1.136 up, *p* < 0.03; **Figure 3A**; Supplementary Table S3). Additionally, a transcript encoding both ubiquitin and nickel superoxide dismutase (NiSOD) domains (comp24219_c0) were downregulated on days 19 (1.304 down, *p* < 0.04) and 28 (1.197 down, *p* < 0.005; **Figure 3A**; Supplementary Table S3). A further three transcripts encoding NiSODs and two transcripts encoding MnSODs were annotated (Supplementary Table S5), though no significant expression changes were detected under the conditions used. Two transcripts encoding catalase peroxidase (KatG) exhibited significantly increased expressed in thermally stressed cells (comp80428_c0, day 4, 1.215 up, *p* < 0.001 and comp61565_c0, day 28, 1.110 up, *p* < 0.03; **Figure 3A**; Supplementary Table S3). Nine transcripts encoding ascorbate peroxidases (APX), four peroxiredoxin (Prx) genes, 27 thioredoxin (Trx) genes, and 10 glutathione *S*-transferase (GST) transcripts were detected with significantly different expression throughout the thermal stress (**Figure 3A**; Supplementary Table S3).

Transcripts encoding 41 heat shock proteins (HSPs; HSP90, HSP70, HSP20), heat shock transcription factors (HSTF), and molecular chaperones (DNAJ) were detected with differential expression through all time points. In the downregulated data set, nine transcripts encoding HSPs, DNAJs, and heat shock-related proteins (HRPs) were detected with significantly decreased expression at all three time points (**Figure 3A**; Supplementary Table S3). In the upregulated data set, two HSP70 transcripts (comp71407_c0 and comp76430_c0) were detected with significantly increased expression at all time points (**Figure 3A**; Supplementary Table S3). Further, chloroplast targeted HSPs exhibited no difference in expression patterns compared with cytosolic associated HSPs.

DNA damage repair and proteasomal degradation pathways were differentially regulated in thermally stressed *Symbiodinium* cells. Nine DNA repair RAD proteins (RAD5, RAD16, and RAD23-1) were annotated with differential expression over the course of the experiment (**Figure 3B**; Supplementary Table S3). A single RAD50 DNA repair transcript (comp72013_c0) displayed significantly increased expression at all time points (**Figure 3B**; Supplementary Table S3). DNA photolyases (PHR) and cryptochrome transcripts [cryptochrome DASH (CRYD)] and cryptochrome 2 (CRY2) showed varied expression, six transcripts were detected with significantly increased expression, and six transcripts displayed significantly decreased expression over the experiment (**Figure 3B**; Supplementary Table S3). Ubiquitin proteasome pathway (UPP) components were detected with significant fold changes, including ubiquitination enzymes for conjugation (E1, E2, and E3 enzymes) and deubiquitination (DUBs) and ubiquitin-like modifiers (SUMO, NEDD8, ISG15, APG8, and APG12; Supplementary Table S4). Though a large number (>260) of UPP enzymes and modifiers displayed significant fold changes (Supplementary Table S4), only five transcripts were detected with significantly decreased expression at all time points and 15 transcripts displayed significantly increased fold change at all time points (**Figure 3B**; Supplementary Table S3).

Apoptosis-like transcripts were detected in thermally stressed *Symbiodinium* cells. Seventeen transcripts encoding three metacaspase 1 isoforms (MCA1, MCA1A, and MCA1B) were detected with differential expression in thermally stressed cells (**Figure 3B**; Supplementary Table S3). Three detected metacaspase contigs (two MCA1 isoforms and one MCA1B isoform) were significantly downregulated at all time points (**Figure 3B**; Supplementary Table S3). One transcript (comp71271_c0) encoding a MCA1A isoform displayed significantly increased expression at all time points (**Figure 3B**; Supplementary Table S3). A further 13 metacaspase transcripts were detected with differential expression over the experiment, 10 transcripts were significantly upregulated at at least one time point, and three were significantly downregulated at at least one time point (FDR < 0.05; **Figure 3B**; Supplementary Table S3). Six transcripts encoding apoptosis-inducing factor homologs (AIFB and AIFM3) were detected, one AIFM3 transcript and three AIFB transcripts displayed increased gene expression and two displayed decreased expression across the experiment though not all time points were significantly different to controls (**Figure 3B**; Supplementary Table S3).

Transcripts encoding anti-apoptosis proteins were also differentially expressed in thermally stressed cells. Three transcripts encoding inhibitors of apoptosis (IAP; Baculoviral IAP repeat-containing protein isoforms) were detected with increased expression on day 4 (BIRC2; comp13582_c0, 1.596 up, *p* < 0.02) and day 19 (BIRC3; comp12814_c0, 1.862 up, *p* < 0.04 and comp40288_c0, 1.482 up, *p* < 0.02; **Figure 3B**; Supplementary Table S3). One further apoptosis inhibitor 1 (IAP1, comp115062_c0) displayed decreased expression on day 19 (1.183 down, *p* < 0.02) and day 28 (1.197 down, *p* < 0.03; **Figure 3B**; Supplementary Table S3). Transcripts encoding suppressors of apoptosis, Bax inhibitor 1 (BI1) family genes, were detected in the *Symbiodinium* transcriptome including 13 protein lifeguard isoforms (LFG1–LFG4; **Figure 3B**; Supplementary Table S3). In thermally stressed cells nine BI1 family genes were detected with differential expression including three LFG1, three LFG2, two LFG3, and a BI1-like protein (**Figure 3B**; Supplementary Table S3). Interesting three transcripts encoding the LFG4 isoform did not exhibit altered expression under the conditions used (Supplementary Table S5).

### Photosynthesis Related Genes

**FIGURE 4:**
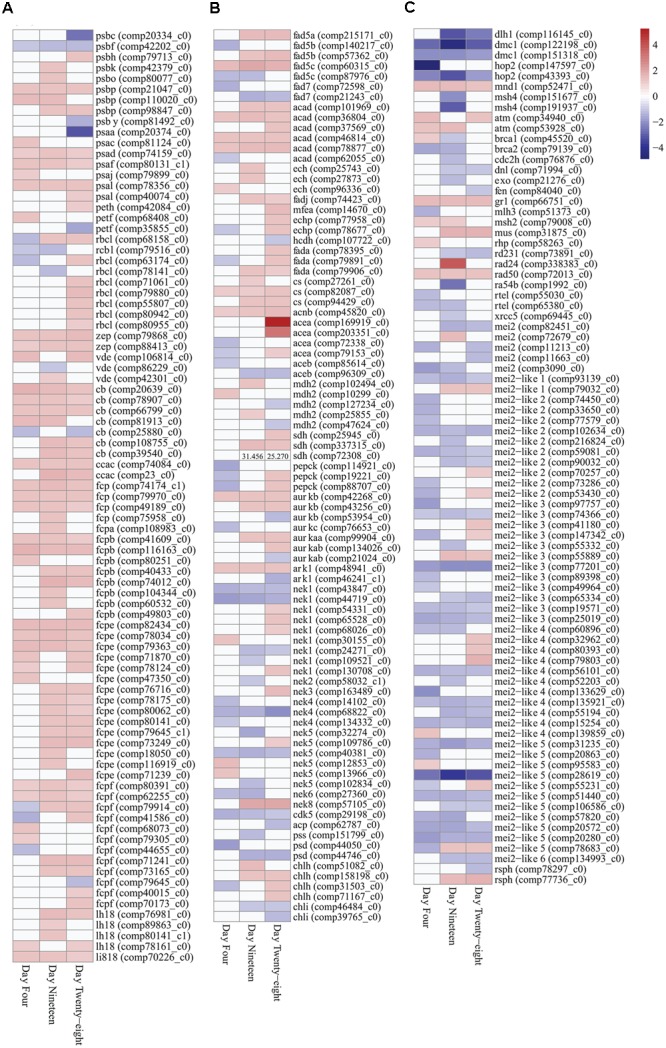
**Expression heatmaps of differentially expressed photosynthesis, metabolism and growth genes (FDR < 0.05) in *Symbiodinium* after exposure to thermal stress for 4, 19, and 28 days.** Data are expressed as fold-changes relative to control; only significant data are shown (*p* < 0.05), non-significant data denoted as white boxes. **(A)** Differential expression of photosynthesis related genes. **(B)** Differential expression of fatty acid desaturases, fatty acid β-oxidation enzymes, glyoxylate cycle enzymes, selected serine/threonine-protein kinases, and cellular component biosynthesis genes. High expression levels of a SDH transcript are denoted numerically. **(C)** Differential expression of meiosis-specific, meiosis-related, and RNA binding proteins. Abbreviations: psb, photosystem II protein; psa, photosystem I protein; peth, ferredoxin-NADP+ reductase; petf, ferredoxin; rbcl, ribulose-1,5-bisphosphate carboxylase/oxygenase; zep, zeaxanthin epoxidase; vde, violaxanthin de-epoxidase; cb, chlorophyll binding protein; ccac, caroteno-chlorophyll *a-c* binding protein; fcp, fucoxanthin-chlorophyll *a-c* binding protein; lh18, light-harvesting complex I protein; li818, chlorophyll *a-b* binding protein l1818; fad, delta-fatty acid desaturase; acad, acyl-CoA dehydrogenase; ech, enoyl-CoA hydratase; fadj, fatty acid oxidation complex subunit; mfea, peroxisomal multifunctional enzyme a; echp, peroxisomal bifunctional enzyme; hcdh, 3-hydroxyacyl-CoA dehydrogenase; fada, β-ketothiolase; cs, citrate synthase; acnb, aconitase; acea, isocitrate synthase; aceb, malate synthase; mdh2, malate dehydrogenase; sdh, succinate dehydrogenase (ubiquinone) flavoprotein subunit; pepck, phosphoenolpyruvate carboxykinase; aurk, aurora kinase; ark, aurora-related kinase; nek, never-in-mitosis A-related kinase; cdk, cyclin-dependent kinase; acp, acyl carrier protein; pss, CDP-diacylglycerol-serine *O*-phosphatidyltransferase; psd, phosphatidylserine decarboxylase proenzyme; chl, magnesium-chelatase subunit; atm, serine/threonine-protein kinase atm; brca, breast cancer susceptibility homolog; cdch2, cell division control protein; dlh1, meiotic recombination protein; dmc1, meiotic recombination protein; dnl, DNA ligase; exo, exonuclease; fen, flap endonuclease; gr1, protein gamma response 1; hop2, homologous-pairing protein 2 homolog; mei2, meiosis protein; mei2-like, meiosis protein-like protein; mlh, DNA mismatch repair protein; mnd, meiotic nuclear division protein; msh, muts protein homolog; mus, crossover junction endonuclease; ra, DNA repair and recombination protein; rad24, DNA damage checkpoint protein; rad50, DNA repair protein; rd, DNA repair protein; rsph, radial spoke head homolog; rtel, regulator of telomere elongation helicase; xrcc, x-ray repair cross-complementing protein. Heatmaps generated using the ‘pheatmap’ package.

Nine transcripts encoding polypeptide subunits of PS II (*psbC, psbF, psbH, psbK, psbO, psbP*, and *psbY*) were detected with significant changes in expression (**Figure 4A**). Genes encoding PS II D1 protein, PS II D2 protein, and CP47 reaction center protein (encoded on plastid mini-circles by *psbA, psbD*, and *psbB*, respectively) were annotated in the transcriptome (Supplementary Table S5), though no significant fold changes were detected under the experimental conditions used (FDR < 0.05). One transcript encoding *psbF* (comp42202_c0), which encodes cytochrome b559 β forming part of the reaction center core of PS II, displayed significant downregulation at all time points (**Figure 4A**). Seven transcripts encoding polypeptide subunits of PS I (*psaA, psaC, psaD, psaF, psaJ*, and *psaL*) were detected with significant changes in expression (**Figure 4A**). Plastid minicircle genes encoding integral membrane peptide subunits of PS I were annotated in the transcriptome, expression of the PS I P700 chlorophyll *a* apoprotein A1 gene (*psaA*) were decreased at day 28 (3.306 down fold change, *p* < 0.02; **Figure 4A**), no significant differences in expression were detected for the PS I P700 chlorophyll *a* apoprotein A2 (*psaB*; Supplementary Table S5). Differential expression of transcripts encoding ferredoxin-NADP+ reductase [*petH* (comp42084_c0)] and ferredoxin [*petF* (comp68408_c0 and comp35855_c0)] were detected with significant differences in expression under thermal stress (**Figure 4A**; Supplementary Table S3).

Plastid-targeted genes were differentially expressed in thermally stressed *Symbiodinium* cells. For example nine transcripts encoding the unique Form II ribulose 1,5-bisphosphate carboxylase/oxygenase (RuBisCo) enzyme (*rbcL*, large subunit) were differentially expressed under thermal stress (**Figure 4A**; Supplementary Table S3). Expression of RuBisCo transcripts were temporally varied, six isoforms displayed significantly increased expression at day 28 (largest 1.465 up fold change). Additionally, a single RuBisCo isoform (comp68158_c0) with significant differential expression at all time points were identified (**Figure 4A**; Supplementary Table S3).

Genes involved in photoprotective mechanisms in *Symbiodinium* including NPQ were differentially expressed under thermal stress. In *Symbiodinium*, the xanthophyll cycle involves the de-epoxidation and epoxidation reactions of diadinoxanthin/diatoxanthin for energy dissipation and to regulate the amount of energy reaching the photosystem reaction centers. Three violaxanthin de-epoxidase (*vde*) transcripts were detected with significant changes in expression at time points throughout the thermal stress (**Figure 4A**; Supplementary Table S3). Two zeaxanthin epoxidase (*zep*) transcripts (comp79868_c0 and comp88413_c0), displayed significantly increased fold changes through all time points (**Figure 4A**; Supplementary Table S3).

Expression of the nuclear-encoded LHC proteins responsible for enhancing light transfer to core photosystems and photoprotection were determined in thermally stressed cells. A transcript encoding the extrinsic water-soluble LHC peridinin-chlorophyll *a*-*b*inding protein (PCP) was detected in the *Symbiodinium* transcriptome (comp80938_c0; Supplementary Table S5), though no significant fold changes were detected under the experimental conditions used (FDR < 0.05). Fifty-four transcripts encoding the *Symbiodinium* specific intrinsic membrane-bound LHC isoforms (acpPC) were detected with significant fold changes during the thermal stress (PFAM ID PF00504.16). Blast annotations shown, (cb, chlorophyll binding protein; ccac, caroteno-chlorophyll *a*-*c* binding protein; fcp, fucoxanthin-chlorophyll *a*-*c* binding protein; lh18, LHC I protein; li818, chlorophyll *a*-*b* binding protein l1818; **Figure 4A**; Supplementary Table S3). Of these 54 intrinsic LHC transcripts, 14 were detected with significant fold changes at all time points (13 were upregulated and one transcript displayed mixed expression; **Figure 4B**). The remaining 40 light-harvesting protein complex transcripts detected displayed predominately increased expression at all time points (85% up fold change), though not all were significantly different to controls (**Figure 4A**; Supplementary Table S3). Additionally, a further 29 transcripts annotated in the transcriptome as light-harvesting protein complexes displayed no change in expression under the experimental conditions (Supplementary Table S5).

### Metabolism and Growth

Genes encoding enzymes for fatty acid desaturation and fatty acid β-oxidation were detected with differential expression in *Symbiodinium* cells exposed to thermal stress. Seven delta-fatty acid desaturase transcripts were detected with differential expression in thermally stressed cells (**Figure 4B**). Five contigs were annotated as delta-5 desaturases (isoforms fad5A, fad5B, and fad5C) and two were annotated as palmitoyl-monogalactosyldiacylglycerol delta-7 desaturase. Throughout the experiment two palmitoyl-monogalactosyldiacylglycerol delta-7 desaturase (fad7) contigs were downregulated (comp72598_c0, day 4, 1.399 down, *p* < 0.04; comp21243_c0, day 19, 1.565 down, *p* < 0.01, day 28, 1.210 down, *p* < 0.01) whereas, three of the delta-5 desaturase contigs were significantly upregulated at days 19 and 28 (**Figure 4B**; Supplementary Table S3). Representatives of all genes involved in the β-oxidation of fatty acids were differentially expressed over the course of the experiment. Six transcripts encoding acyl-CoA dehydrogenases (ACADs) were detected with differential expression over the course of the experiment, with five displaying significantly increased fold changes on day 28 (**Figure 4B**). Transcripts encoding enoyl-CoA hydratases (three ECH), fatty acid oxidation complex subunit alpha (one FADJ), and peroxisomal bifunctional enzymes (1 MFEA and 2 ECHP) were all detected with increased expression in thermally stressed cells (**Figure 4B**; Supplementary Table S3). One transcript, encoding a probable 3-hydroxyacyl-CoA dehydrogenase (HCDH), was detected with decreased expression on day 28 (comp107722_c0, 1.102 down, *p* < 0.02; **Figure 4B**). Three transcripts encoding β-ketothiolases (FADA), responsible for the final cleavage step of the β-oxidation pathway were detected with significantly increased expression on day 28 (**Figure 4B**). One of the β-ketothiolases (comp79906_c0) was annotated as a peroxisomal associated transcript, and displayed significantly increased expression on day 19 (1.276 up, *p* < 0.01) and day 28 (1.169 up, *p* < 0.03; **Figure 4B**). Further, three transcripts encoding peroxisome membrane proteins (PMP34), two peroxisome adenine nucleotide carrier isoforms (PNC1 and PNC2), and two peroxisome ATP-binding cassette sub-family D members were all detected with significant increases in expression under these conditions (Supplementary Table S5).

Glyoxylate cycle and gluconeogenic pathway enzymes were detected with differential expression in thermally stressed cells. Transcripts encoding enzymes of the glyoxylate cycle including citrate synthase (CS), aconitase (acnB), isocitrate lyase (aceA), malate synthase (aceB), and malate dehydrogenase (MDH2) were detected with differential expression in thermally stressed *Symbiodinium* cells (**Figure 4B**). Specifically, four transcripts encoding isocitrate lyases (aceA) were detected with significant changes in expression under thermal stress (**Figure 4B**; Supplementary Table S3). Two of the isocitrate lyase contigs displayed decreased expression on day 4, whereas on day 28 three of the isocitrate lyase contigs displayed significantly increased fold change compared to controls (**Figure 4B**). Two malate synthase (aceB) transcripts were detected with decreased expression, comp85614_c0 on day 4 (1.171 down, *p* < 0.04) and comp96309_c0 on day 19 (1.259 down, *p* < 0.002), and day 28 (1.263 down, *p* < 0.008; **Figure 4B**). Three transcripts encoding succinate dehydrogenase flavoprotein subunits were also detected with significantly increased expression (comp25945_c0 on day 28, 1.294 up, *p* < 0.009), and comp72308_c0 on day 19, 1.433 up, *p* < 0.02 and day 28, 1.287 up, *p* < 0.004, and comp337315_c0 on day 19, 31.456 up, *p* < 0.02 and day 28 25.270 up, *p* < 0.03 (**Figure 4B**). In addition, three contigs encoding phosphoenolpyruvate carboxykinase (PEPCK) were detected with differential expression in thermally stressed cells (**Figure 4B**; Supplementary Table S3).

Serine/threonine-protein kinases are crucial components of diverse signaling pathways and for regulation of cell proliferation, meiosis, and apoptosis. One hundred and seventy-seven transcripts representing more than 20 serine/threonine-protein kinase families were detected with significant changes in expression in thermally stressed cells (Supplementary Table S3). Nine transcripts encoding three classes of aurora kinases [Aurora-A, Aurora-B, and Aurora-C (AurK)] and two aurora-related kinases (ARKs) were detected with significant fold changes throughout the experiment (**Figure 4B**; Supplementary Table S3). Twenty-two never-in-mitosis A serine/threonine kinase (Nek) transcripts representing seven Nek families were detected with differential expression (**Figure 4B**; Supplementary Table S3). One transcript encoding a cyclin-dependent kinase (CDK5, comp29198_c0) was detected with significantly decreased expression at all time points (**Figure 4B**; Supplementary Table S3).

Differential regulation of cellular component biosynthesis were detected in thermally stressed *Symbiodinium* cells. Transcripts encoding lipid biosynthetic acyl carrier protein (*acp*; comp62787_c0), CDP-diacylglycerol-serine *O*-phosphatidyltransferase (*pss*; comp44746_c0), and two phosphatidylserine decarboxylase proenzymes (*psd*) exhibited decreased expression in thermally stressed cells (**Figure 4B**; Supplementary Table S3). Four magnesium-chelatase subunit H (*chlH*) transcripts were detected with significantly increased expression over the experiment, whereas, two magnesium-chelatase subunit I (*chlI*) transcripts exhibited decreased expression on day 28 (comp46484_c0, 1.173 down, *p* < 0.001 and comp39765_c0, 1.181 down, *p* < 0.05; **Figure 4B**; Supplementary Table S3). Additionally, one transcript encoding a magnesium-chelatase subunit D (*chlD*; comp84001_c0; Supplementary Table S5), displayed no changes in expression under the conditions used here (FDR < 0.05).

Meiosis-specific and meiosis-related genes previously annotated in *Symbiodinium* (Chi et al., 2014; Levin et al., 2016) were detected in the transcriptome and in the repertoire of differentially expressed transcripts. Eight meiosis-specific transcripts representing four genes (three *Dmc1*, two *Hop2*, one *Mnd1*, and two *Msh4)* were annotated with differential expression in thermally stressed cells (**Figure 4C**; Supplementary Table S3). Twenty meiosis-related transcripts representing 17 genes were also annotated with differential expression in thermally stressed *Symbiodinium* cells (**Figure 4C**; Supplementary Table S3). Additionally, 52 transcripts encoding protein MEI2 and MEI2-like isoforms were detected with differential expression (**Figure 4C**; Supplementary Table S3). MEI2 genes have been annotated in *Schizosaccharomyces pombe* and MEI2-like proteins have been annotated in *Arabidopsis thaliana* and *Oryza sativa* subsp. *japonica*. Two radial spoke head homologs (*Rsph1*) were detected with differential expression (**Figure 4C**; Supplementary Table S3), previously detected around the chromosomes during metaphase in male gametes undergoing meiotic division. However, *Rsph1* has also been implicated in axenomal central pair regulating dynein activity for flagella and cilia movement.

## Discussion

Predictions for future climate conditions estimate the sea-surface temperature to rise between 1.1 and 6.4°C depending on emissions scenarios (Solomon et al., 2007). The implications of this change for coral reefs and the marine ecosystems they support are unknown. However, this prolonged exposure of coral reefs to elevated temperatures may have catastrophic consequences and result in the loss of many species. Through examining the physiological response of *Symbiodinium* cultures to elevated temperatures, and linking this to molecular processes that are altered under thermal stress we may begin to understand how reefs may be affected in the future. In this study, we exposed cultured *Symbiodinium* sp. (clade F) to thermal stress (28 days, ∼30–31.5°C; **Figure 1A**) and generated a library of contigs that represent the transcriptome under future temperature conditions.

Analysis of the *Symbiodinium* transcriptome revealed more than 37% were differentially expressed under thermal stress (FDR < 0.05). A large number of DEGs (23,654 unique contigs) were detected, of which 2,798 were differentially expressed at all time points (**Figure 2A**). The majority of DEGs [21,878 genes (92.49%)], displayed a ≤2-fold change in expression. This is reflective of previous targeted expression studies in *Symbiodinium* where relatively small changes in gene expression were determined (Leggat et al., 2011a; Rosic et al., 2011; McGinley et al., 2012; Ogawa et al., 2013; Gierz et al., 2016), due to this it has been hypothesized that translational or post-translational regulation may be critical in *Symbiodinium* cellular responses. Biological process GO visualization revealed that distribution of combined time point DEGs (2,798 transcripts) were relatively even in assigned terms between those up and downregulated transcripts (Supplementary Figure S4). Under these conditions regulation of molecular processes in this *Symbiodinium* strain is variable, with components of many pathways exhibiting dissimilar expression patterns.

Over the course of the experiment temperature significantly effected *Symbiodinium* density and photosynthetic efficiency. Previously, growth rates of six cultured *Symbiodinium* strains (representing clades A, B, C, D, and F) with various thermal tolerances where determined at different temperatures (25, 30, and 33°C; Karim et al., 2015). Showing that increased temperatures can alter growth rates and photosynthetic efficiency differently, resulting in the classification of three categories depending on the thermal tolerance of the strain (Karim et al., 2015). In this study, *Symbiodinium* cell density were decreased from day 4 compared to controls (**Figure 1B**), and the photosynthetic ability of thermally stressed cells were maintained until day 14 (**Figure 1C**). The slight but significantly depressed dark-adapted yield (day 14, 19, and 28; **Figure 1C**) indicates that cells exposed to elevated temperatures were exhibiting altered photosynthetic efficiencies in response to thermal stress. However, as complete loss of photosynthetic efficiency did not occur (**Figure 1C**), this strain is categorized as photosynthetically tolerant though growth response were highly sensitive to elevated temperature (Karim et al., 2015).

Analysis of chlorophyll pigments in *Symbiodinium* found significantly increased chlorophyll content in a manner seen previously (McBride et al., 2009; Ogawa et al., 2013; Gierz et al., 2016). Chlorophyll *a* concentration were significantly higher in thermally stressed cells on days 19 and 28 (**Figure 1F**). Comparison of growth rates and chlorophyll content in *Symbiodinium californium* found cultures exhibiting low growth rates (incubated at 5, 10, and 30°C) also showed an increased chlorophyll *a* content, whereas those actively growing had reduced chlorophyll *a* content (McBride et al., 2009). In phytoplankton, variation of chlorophyll *a*-specific absorption has been attributed to packaging of chlorophylls within different pigment-protein complexes (chlorophyll *a*-chlorophyll c-peridinin (ACP) versus PSI; Bissett et al., 1997). It is possible that the increased chlorophyll *a* content observed in thermally stressed *Symbiodinium* cells may be due to changes in the specific pigment-protein complexes within the chloroplasts. Analysis of chlorophyll *c* content (**Figure 1G**) and the ratio of chlorophyll *c* to chlorophyll *a* (**Figure 1H**) found that there were no significant differences between controls and thermally stressed cells. Biosynthesis of chlorophyll involves the ATP-dependent insertion of a magnesium ion into protoporphyrin IX, catalyzed by three magnesium-chelatase subunits (Von Wettstein et al., 1995). Six transcripts encoding two of the three magnesium-chelatase subunits (two *chlI* and four *chlH*) were differentially expressed in thermally stressed cells (**Figure 4B**; Supplementary Table S3). However, expression of these subunits were not consistent with *chlI* subunits detected with significant decreased fold changes, no changes detected in a *chlD* subunit (Supplementary Table S5), and four *chlH* subunit with significantly increased expression during exposure to increased temperatures (**Figure 4B**; Supplementary Table S3). The implications for the variable expression of these subunits critical for chlorophyll biosynthesis requires further investigation.

Measurements of photosynthetic ability are often employed as indicators of *Symbiodinium* cell health (Buxton et al., 2012; Hill and Takahashi, 2014). In this study, Induction Curve + Recovery kinetic recording type were used to determine the ability of cells to respond to light stress. Significant decreases in effective quantum yield at the end of the induction phase were detected on day 4 (**Figure 1D**) and may be indicative of an early photosynthetic response to elevated temperatures. Throughout the remainder of the experiment no significant difference in effective quantum yield between controls and thermally stressed cells were detected after the recovery phase, though values were slightly depressed in treatment cells (**Figure 1D**). NPQ were found to be significantly decreased in treated cells on day 19, and may indicate that the photoprotective mechanisms of cells were impacted by exposure to thermal stress (**Figure 1E**). Though little changes were observed in NPQ, we detected xanthophyll cycle enzyme genes (VDE and ZEP) with significantly different expression (**Figure 4A**; Supplementary Table S3). VDE and ZEP are responsible for the epoxidation and de-epoxidation of dinoxanthin/diadinoxanthin as a photoprotective mechanism by dissipating excess energy.

### Differential Expression of the *Symbiodinium* Antioxidant Network

Photobleaching in *Symbiodinium* induced by thermal stress and high solar irradiance has been linked to oxidative damage resulting from the production of reactive oxygen species (ROS; Murata et al., 2007; Takahashi et al., 2008; Krueger et al., 2014). ROS can be deleterious to cells resulting in oxidative damage to lipids, proteins, and DNA but may also function as second messengers for signal transduction (Lesser, 2006). Defenses against ROS in *Symbiodinium* and other photoautotrophs include an antioxidant network of enzymes such as SODs, catalases, and peroxidases as well as non-enzymatic antioxidants such as glutathione, Trx, Prx, and carotenoids (via the xanthophyll cycle; Lesser, 2006; Bayer et al., 2012; Krueger et al., 2014, 2015). Comparison of *Symbiodinium* types has shown that the antioxidant network and the antioxidant response to thermal stress can vary between strains of different thermal tolerance with implications for photosynthesis and cell viability (Krueger et al., 2014). Within *Symbiodinium* cells exposed to thermal stress transcripts encoding CuZnSOD, MnSOD, KatG, and ZEP were all significantly upregulated whereas, expression of APX and VDE transcripts varied (**Figures 3A** and **4A**; Supplementary Table S3). Within the *Symbiodinium* transcriptome assembly, three transcripts encoding prokaryotic-like NiSOD and one transcript encoding both ubiquitin and NiSOD domains were also identified. Genes containing ubiquitin/NiSOD and NiSOD domains have also been identified in the antioxidant gene repertoire of *Symbiodinium* sp. CassKB8 and *Symbiodinium* sp. Mf1.05b (Bayer et al., 2012) and two C1 type *Symbiodinium* (Levin et al., 2016) and may represent genes acquired from prokaryotes by lateral gene transfer. Within *Symbiodinium* sp. CassKB8 and *Symbiodinium* sp. Mf1.05b a high number of Trx domain containing genes [106 and 73 Trx genes, respectively (PF00085.14)] were identified (Bayer et al., 2012), within this transcriptome assembly 85 Trx domain containing genes were identified with 27 Trx genes differentially expressed under thermal stress (**Figure 3A**; Supplementary Table S3). Trx superfamily proteins (Trx and Trx-like proteins) have roles in the oxidative stress response by regulating the redox state, aid in the repair of damaged photosystems (Nishiyama et al., 2011) and regulate many photosynthetic enzymes in plants (including Calvin cycle enzymes such as glyceraldehyde 3-phosphate dehydrogenase, phosphoribulokinase, and RuBisCo activase; Hisabori et al., 2007). Differential expression of components of the antioxidant network implicated in protecting cells from ROS and regulating photosynthetic processes were detected here in thermally stressed *Symbiodinium*. Given that we did not observe a loss in photosynthetic ability of cells, but did observe a shift in metabolism, and don’t know the mechanism of regulation of RuBsiCo form II in *Symbiodinium* the effect of cell redox state on photosynthesis and CO_2_ fixation via the Calvin cycle under thermal stress requires further investigation.

### Cell Cycle in Thermally Stressed *Symbiodinium*

The life cycle of *Symbiodinium* has predominately been considered asexual, deduced by studying morphological transitions and direct observations of mitotic growth. During the vegetative growth phase, haploid cells undergo a diel cycle of mitosis (Fitt and Trench, 1983; Santos and Coffroth, 2003). Progression of the cell cycle in *Symbiodinium* has been shown to halt when fatty acid syntheses were inhibited by addition of cerulenin [interpreted from decreased free fatty acid and phosphatidylethanolamine (PE) content] (Wang et al., 2013). In prokaryotic and eukaryotic cells, PEs are structural components of membranes and *de novo* synthesis of PE occurs via the CDP-ethanolamine pathway a branch of the Kennedy pathway (Gibellini and Smith, 2010). Mutant *Escherichia coli* cells deficient in PE due to defective CDP-ethanolamine pathway genes (*pss* and *psd)*, showed reduced transcription of flagella genes, resulting in decreased motility and chemotaxis compared to wild type cells (Shi et al., 1993). In this study, components of the CDP-ethanolamine pathway were differentially expressed in thermally stressed cells, one CDP-diacylglycerol-serine *O*-phosphatidyltransferase transcript (encoded by *pss*) displayed significantly decreased expression on day 19 (comp44746_c0) and two phosphatidylserine decarboxylase transcripts (encoded by *psd*) were significantly decreased (comp151799_c0 on day 4 and comp44050_c0 on days 19 and 28; **Figure 4B**; Supplementary Table S3). Therefore, in thermally stressed *Symbiodinium*, decreased expression of CDP-ethanolamine pathway genes for PE synthesis could impact the cell cycle due to reduced glycerophospholipid content available for cellular processes.

Mitotic kinases implicated in cell cycle regulation and DNA damage responses were identified with differential expression in thermally stressed *Symbiodinium.* For example, aurora kinases regulate cell proliferation by controlling M-phase events, such as mitotic spindle attachment (Aurora-A; Nigg, 2001) and NimA-related kinases (Neks) have roles in cell cycle control regulating establishment of the mitotic spindle, chromosome condensation, response to DNA damage, and flagella/cilia development (Nigg, 2001; Fry et al., 2012). Additionally cell division control protein homologs and cyclin-dependent kinases were also detected with differential expression in thermally stressed cells (**Figure 4B**; Supplementary Table S3). Expression of these cell cycle regulators were not consistent across the gene families identified. Of the nine differentially expressed transcripts encoding *nek1* genes in thermally stressed cells, four contigs were significantly increased and three were significantly decreased on day 28 (**Figure 4B**; Supplementary Table S3). Therefore, although a large number of mitotic kinases exhibited differential expression, linking the expression of these cell cycle regulatory proteins in thermally stressed cells to the observed physiological response is not feasible with the current data.

Documentation of *Symbiodinium* sexual recombination has been difficult, as cytological evidence of karyogamy has not been found (Chi et al., 2014). However, investigation of population genetic patterns and advances in genomic data has potentially identified a collection of meiotic genes in *Symbiodinium* (Chi et al., 2014; Wilkinson et al., 2015; Levin et al., 2016). In this study, we identified a number of these meiosis-specific and meiosis-related transcripts (**Figure 4C**; Supplementary Table S3), providing further support for the occurrence of sexual recombination in *Symbiodinium.* Additionally the occurrence of sexual recombination may be restricted to specific conditions, i.e., to free-living symbionts, or be inactivated by symbiotic conditions (Chi et al., 2014), reducing the opportunities for cytological observation. Recently, analysis of the transcriptional response of two type C1 *Symbiodinium* to heat stress (32°C), identified upregulation of two mutS homolog genes (*Msh4* and *Msh5*) and no significant changes in other *Msh* genes (*Msh1, 2, 3*, and *6*), suggesting these genes are essential to meiosis lending support to adaption (Levin et al., 2016). In this study, two transcripts annotated as *Msh4* were significantly downregulated on day 19 (comp151677_c0 and comp191937_c0), one transcript annotated as *Msh2* exhibited significant upregulation on days 4 and 19 (comp79008_c0), whereas, three transcripts annotated as *Msh5* (comp169914_c0, comp201605_c0, and comp32251_c0) and an *Msh6* transcript (comp43641_c0) displayed no change in expression (Supplementary Tables S1 and S2). In this study exposure of *Symbiodinium* to thermal stress resulted in reduced growth rate potentially inducing a cell cycle phase conducive to meiotic-like division. However, differential expression of these genes may also be for repair of DNA damage or the stabilization of DNA in thermally stressed *Symbiodinium*.

Genes encoding RNA binding proteins containing the RNA recognition motif (RRM) were detected with differential expression. In thermally stressed *Symbiodinium* cells, RRM containing genes were detected with similarity to the *MEI2* gene identified in *S. pombe* and the MEI2-like gene families identified in *A. thaliana* and *O. sativa*. In *S*. *pombe*, accumulation and localization of the MEI2 protein to meiRNA results in pre-meiotic DNA synthesis and entry into meiosis I (Anderson et al., 2004; Jeffares et al., 2004). MEI2-like genes have been identified in various eukaryotes including *Chlamydomonas reinhardtii*, with single copies identified in ascomycete fungi, alveolates, and entamoebidae and gene families identified in plants, however, they are not found in metazoans (Anderson et al., 2004; Jeffares et al., 2004). Analysis of conserved orthologs between clade C3 and A1 *Symbiodinium* identified an RNA binding protein MEI2 homolog with hits to the alveolate *Plasmodium falciparum* genome (Voolstra et al., 2009b). Recently, two MEI2-like proteins (MEI2-like 2 and MEI2-like 4) were identified with differential expression between species in the comparison of clade B *Symbiodinium* species (Parkinson et al., 2016). The MEI2-like genes share conserved RRM domains with MEI2 genes (Anderson et al., 2004; Jeffares et al., 2004), though functionally are believed to be involved in cell development during vegetative growth in *A. thaliana* as well as regulating meiosis (Kaur et al., 2006). RNA binding proteins have also been implicated in chromatin organization and remodeling (Kaur et al., 2006) and MEI2-like gene knockout in *Plasmodium yoelii* prevents a post-transcriptional regulatory mechanism inhibiting liver schizont stage maturation (Dankwa et al., 2016). The function of these RNA binding proteins in thermally stressed *Symbiodinium* is unclear, but with 52 transcripts displaying differential expression further studies are needed.

The molecular response to stress can vary with many pathways in place to minimize damage and re-establish cellular homeostasis. The type and degree of stress can ultimately alter the fate of the cell with various cellular functions targeted during stress responses such as cell cycle control, molecular chaperoning, protein repair, protein degradation, and DNA repair, however, if cellular function cannot be regained cell death (apoptosis) may occur (Kültz, 2005). Here within *Symbiodinium* exposed to thermal stress DEGs encoding RAD DNA repair proteins, DNA photolyases, UPP components, molecular chaperones (HSPs and DNAJ), pro-apoptosis (metacaspases and apoptosis-inducing factors) and anti-apoptosis [IAPs and Bax1 (protein lifeguard) were differentially expressed; **Figure 3B**; Supplementary Table S3]. Previous studies of *Symbiodinium* have identified HSPs (HSP70 and HSP90) and HRPs (Leggat et al., 2007; Rosic et al., 2014) and quantified their expression in response to stress (Leggat et al., 2011a; Rosic et al., 2011; Barshis et al., 2014), however, under the various conditions used expression patterns of these molecular chaperones varied. Exposure of *Symbiodinium* to thermal stress, elicited components of stress response pathways detected here using gene expression analysis (**Figures 3A,B**; Supplementary Table S3), which were reflected by decreased cell growth within the physiological parameters measured. By understanding the roles of molecular chaperones in maintaining cellular functions including protein folding and the effect of the proteasomal repair and degradation pathways we may improve our understanding of the stress response of *Symbiodinium* cells.

### Photosynthesis in Thermally Stressed *Symbiodinium*

Understanding of the photosynthetic machinery gene homologs and their organization has recently advanced with sequencing of the *Symbiodinium* chloroplast and nuclear genomes (Shoguchi et al., 2013; Barbrook et al., 2014; Mungpakdee et al., 2014). Previously, characterization of *Symbiodinium* photosystem subunit proteins and genes has examined PS II core proteins *psbA* (D1 protein) and *psbD* (D2 protein), PS II manganese-stabilizing protein (or PsbO protein) encoded by *psbO*, and PS I core protein *psaA* (P_700_ protein; Iglesias-Prieto and Trench, 1997; Warner et al., 1999; Iida et al., 2008; Takahashi et al., 2008; McGinley et al., 2012; Castillo-Medina et al., 2013; Gierz et al., 2016). Photoinhibition of PS II and decreased expression of PS II D1 protein (D1 protein content and *psbA* gene expression) have been observed in thermally stressed *Symbiodinium* cells (Takahashi et al., 2008; McGinley et al., 2012; Gierz et al., 2016). In this study, exposure of *Symbiodinium* to thermal stress resulted in a slight decrease in dark-adapted yield (**Figure 1C**), a measurement of the PSII activity, though PS II core proteins encoded by *psbA* and *psbD* showed no significant changes under these conditions. However, transcripts encoding various subunits of photosystem II (PS II), the cytochrome b_6_f complex, photosystem I (PS I), ATP synthase, cytochrome c_6_, phycocyanin beta, ferredoxin (FRX), and ferredoxin-NADP (+) reductase (FNR) were detected with differential expression under thermal stress conditions (**Figure 4A**; Supplementary Table S3). Notably four transcripts encoding two extrinsic proteins of the PS II oxygen-evolving complex (*psbO* and *psbP*) were significantly upregulated in thermally stressed cells (**Figure 4A**; Supplementary Table S3). PsbO homologs are found in higher plants, green algae, red algae, diatoms and cyanobacteria and stabilize the Mn cluster and may be critical for the recruitment and assembly of PS II (Ifuku and Noguchi, 2016). PsbP homologs have been annotated in plants, green algae, and cyanobacteria, have high calcium ion binding affinity and may aid in stabilizing the PS II-LHC II supercomplexes in higher plants (Ifuku et al., 2011; Ifuku and Noguchi, 2016).

Biosynthesis of photosynthetic complexes is a controlled process relying on synthesis, insertion, and coordination of each subunit for successful assembly (Rochaix, 2011). Assembly of PS I in *C. reinhardtii* relies on the insertion of the scaffold anchor protein PsaB, followed by PsaA forming the chlorophyll *a*-protein complex I and finally by PsaC, after which the other subunits are incorporated (Rochaix, 2011). Photosynthetic ATP synthesis relies on the generation of a proton gradient, either by linear electron flow from PS II to PS I (producing reduced NADP) through the cytochrome *b*_6_f complex or cyclic electron flow (CEF) through PS I and the cytochrome *b*_6_f complex (Rochaix, 2011). Additionally, CEF around PSI in *Symbiodinium* has been demonstrated to increase under short moderate heat stress, proposed to alleviate photoinhibition by dissipating excess energy (qE; Aihara et al., 2016). In this study, expression of the PS I P_700_ gene (*psaA*) varied over the experiment but significantly decreased on day 28 (**Figure 4A**; Supplementary Table S3). Previous targeted studies of *psaA* across multiple strains of *Symbiodinium* also found thermal stress resulted in decreased gene expression (McGinley et al., 2012). This change in *psaA* expression could therefore impair assembly of new PS I complexes in *Symbiodinium* exposed to thermal stress, potentially disrupting photoprotection via CEF and the synthesis of ATP and reduction of NADP, which are required for cellular metabolic processes including carbon fixation via the Calvin cycle.

In *Symbiodinium*, the integral LHC family has been studied due to their proposed functional roles of enhancing light capture and photo-protection by dissipating excess energy (Iglesias-Prieto et al., 1993; Takahashi et al., 2008). Intrinsic LHCs have therefore been implicated in the stress response of *Symbiodinium* cells (Takahashi et al., 2008; Maruyama et al., 2015; Xiang et al., 2015; Gierz et al., 2016). High diversification of the integral light-harvesting gene family (*acpPC*) in *Symbiodinium* has been shown following analysis of the *S. minutum* genome (Maruyama et al., 2015) and analysis of the *Symbiodinium* sp. C3 *acpPC* gene repertoire (Boldt et al., 2012). The expression of *acpPC*s have been characterized in *Symbiodinium* between two strains of varied thermal tolerance (Takahashi et al., 2008), within a coral host under thermal stress (Gierz et al., 2016) and under light stress (Xiang et al., 2015). In this study, we identified 54 differentially expressed transcripts encoding *acpPCs* in thermally stressed cells (Supplementary Table S3), of these LHC genes 14 were significantly expressed at every time point throughout the experiment (**Figure 4B**). The functional associations of *Symbiodinium* LHCs cannot be determined from this study, though the varied expression of *acpPC* genes following exposure to thermal stress may be indicative of specific gene function.

### Fatty Acid Desaturases

Fatty acids have been quantified in *Symbiodinium* in attempts to link thermal tolerance to thylakoid membrane lipid composition (Tchernov et al., 2004) and to develop lipid biomarkers for stress (Kneeland et al., 2013). Analysis of lipid content has shown that thermally tolerant species possess different ratios of polyunsaturated fatty acid (PUFA) compared to thermally sensitive strains (Tchernov et al., 2004). However, in multiple clades the lipid composition of whole cells versus enriched chloroplast fractions (Díaz-Almeyda et al., 2011) and the lipid profile (Kneeland et al., 2013) has shown that PUFA desaturation cannot be used to estimate thermal sensitivity of *Symbiodinium*. Fractionation of chloroplasts has shown that between clades lipid composition can differ, and desaturation and isomerization of these fatty acids can alter the melting point of thylakoid membranes, with increased membrane fluidity observed in thermally stressed *S. microadriaticum* A1 (Díaz-Almeyda et al., 2011). Further studies of *Symbiodinium* sp. type C1 and subtype D1 identified decreases in the desaturation ratio and in the fatty acid-to-sterol ratio in cells incubated above 30°C (Kneeland et al., 2013). However, as total fatty acids were saponified, the sources of change in the lipid profile (storage lipids versus membrane lipids) could not be discerned (Kneeland et al., 2013). In this study *Symbiodinium* lipid content were not quantified, so the lipid profiles cannot be estimated. However, DEGs encoding fatty acid desaturase were detected in thermally stressed *Symbiodinium* cells (**Figure 4B**; Supplementary Table S3). Recently, transcriptome analysis of multiple *Symbiodinium* clade B strains revealed differences in expression of fatty acid metabolism and biosynthesis pathway genes potentially related to membrane composition, energy storage, and varied growth rates between species (Parkinson et al., 2016).

Previously, in clades C and D type *Symbiodinium*, orthologs of the palmitoyl-monogalactosyldiacylglycerol delta-7 desaturase were annotated in each assembly, with significantly elevated d_N_/d_s_ along the clade D lineage (Ladner et al., 2012). As described in the clades C and D analysis, these orthologs are very similar to the palmitoyl-monogalactosyldiacylglycerol delta-7 desaturase (ADS3) in *A. thaliana* which are involved in the desaturation of hexadecatrienoic acid (16:3Δ^7,10,13^) (Ladner et al., 2012). As mentioned previously, the lipid composition of thylakoids determined that the ratios of fatty acids (C16, C18, and C22) can change under thermal stress influencing thylakoid membrane fluidity (Díaz-Almeyda et al., 2011). In this study, two classes of fatty acid delta desaturases involved in PUFA biosynthesis were detected with differential expression in *Symbiodinium* exposed to thermal stress (**Figure 4B**). Without having quantified the lipid content of fractionated thylakoids in this study we cannot identify if the increased delta-5 desaturase activity and decreased delta-7 desaturase activity were specific for membrane lipids or storage lipids. Though the differential expression of fatty acid desaturase enzymes detected has not previously been reported, and future studies in *Symbiodinium* may benefit from pairing assays of both fatty acid desaturase expression and lipid content quantification.

### Lipid Catabolism in Thermally Stressed *Symbiodinium*

Analysis of DEGs detected multiple transcripts encoding the four enzymes of the β-oxidation pathway and two enzymes of the glyoxylate cycle (**Figure 4B**). DEGs encoding the four enzymes (acyl-CoA dehydrogenase, enoyl-CoA hydratase, HCDH, and β-ketothiolase) of the fatty acid β-oxidation pathway (**Figure 4B**; Supplementary Table S3) were detected in thermally stressed *Symbiodinium*. Additionally enzymes targeted for both mitochondrial and peroxisomal β-oxidation were annotated, in mammalian cells β-oxidation occurs in both the mitochondria and peroxisomes, whereas, in plant cells β-oxidation is restricted to peroxisomes (Poirier et al., 2006). The peroxisomal β-oxidation pathway in mammalian cells is used for very-long-chain fatty acids and their derivatives that are otherwise slowly degraded in the mitochondria (Poirier et al., 2006), and in plants has been linked to the metabolism of fatty acids from membrane lipids supplying acetyl-CoA to the glyoxylate cycle (Cornah and Smith, 2002). As mentioned previously, fatty acid content of *Symbiodinium* sp. C1 and *S. microadriaticum* A1 thylakoid membranes were significantly modified under thermal stress (Díaz-Almeyda et al., 2011), it is possible that in this study we are detecting DEGs of the β-oxidation pathway for the modification of membrane lipid content by removing free fatty acids in thermally stressed cells or the use of storage lipids.

In plants, bacteria, protists, and fungi degradation of fatty acids by β-oxidation generates acetyl-CoA, which may then enter the glyoxylate cycle to produce substrates for gluconeogenesis (Cornah and Smith, 2002). Glyoxylate cycle enzymes have been identified in yeast and plants under starvation (Cornah and Smith, 2002), in a number of corals from genome and transcriptome analyses (Meyer et al., 2009; DeSalvo et al., 2010; Kenkel et al., 2013; Shinzato et al., 2014) in dinoflagellates (*Karenia brevis* and *Amphidinium carterae*; Bachvaroff et al., 2004; Butterfield et al., 2013), and recently in the *S. kawagutii* genome (Lin et al., 2015). The glyoxylate cycle in plants and filamentous fungi occurs in glyoxysomes, which have also been identified in *Symbiodinium* by transmission electron microscopy (Sammarco and Strychar, 2013). It is also possible that the peroxisomal β-oxidation transcripts identified may instead be glyoxysomal-associated enzymes in *Symbiodinium*. Two enzymes of the glyoxylate cycle, isocitrate lyase (aceA) and malate synthase (aceB; Kornberg and Madsen, 1958) were detected with differential expression in thermally stressed *Symbiodinium* (**Figure 4B**; Supplementary Table S3). Three of the four differentially expressed transcripts encoding isocitrate lyase orthologs were significantly upregulated on day 28 (**Figure 4B**), this enzyme cleaves isocitrate into glyoxylate and succinate (Kornberg and Madsen, 1958). However, the contigs encoding malate synthase were downregulated in thermally stressed cells (**Figure 4B**; Supplementary Table S3). In gluconeogenesis, the conversion of malate/oxaloacetate to phosphoenolpyruvate is catalyzed by PEPCK (Pilkis and Granner, 1992). In thermally stressed *Symbiodinium* cells we detected contigs encoding orthologs of *Dictyostelium discoideum* and *Cucumis sativus* PEPCKs (**Figure 4B**; Supplementary Table S3). Of the three PEPCKs annotated all displayed significantly decreased expression on day 4 and the two *D. discoideum* orthologs were later significantly upregulated on day 28 (**Figure 4B**; Supplementary Table S3), indicating that the gluconeogenic pathway were in use in thermally stressed *Symbiodinium* on day 28. This shift to gluconeogenic metabolism could be a mechanism to reduce free fatty acids in cells from membrane remodeling or potentially be indicative of mobilization of fatty acid stores due to inhibition of photosynthesis induced by thermal stress.

Success of the coral-dinoflagellate symbiosis is largely reliant on the exchange of metabolic products (Davy et al., 2012). Though still unclear due to the difficulties of studying the symbiotic relationship, the translocation of photosynthates from symbionts to host cells (including glycerol, glucose, alanine, and organic acids such as citrate, succinate, fumarate, malate, and glycolate) is estimated to account for up to 60% of photosynthetically fixed carbon (Davy et al., 2012). Although the behavior of isolated *Symbiodinium* can vary from that of cells in symbiosis (Sutton and Hoegh-Guldberg, 1990; Davy et al., 2012), the implications of altered metabolism from exposure to thermal stress may ultimately influence the stability of the symbiotic relationship.

This study provides an overview of the transcriptome response of *Symbiodinium* exposed to thermal stress and highlights differential expression of key genes. This study is the first of its kind to employ a moderate thermal stress regime, for a period of 28 days reflective of future temperature conditions, and provides an assessment of physiological parameters that are paired with RNA-Seq analysis. Our results provide a basis for further studies as the transcriptome analysis provides documentation of differentially expressed genes in *Symbiodinium* exposed to thermal stress for an extended time period. Surprisingly, despite small fold changes a large proportion (23,654 genes) of the transcriptome exhibited altered expression. The longitudinal approach used here has also allowed us to identify genes that display consistently altered expression and those that are only transiently differentially expressed. Differential expression of key stress, photosynthesis, metabolism, and cell cycle genes were detected. Differential expression of glyoxylate cycle enzymes reported here, represents the first instance of this in *Symbiodinium*. The implications for the change in *Symbiodinium* metabolism under extended thermal stress and the effect this may have on *Symbiodinium*-host interactions is unknown, though future studies investigating impacts of extended thermal stress should aim to incorporating metabolomics.

## Author Contributions

SG and WL designed thermal stress experiment. SG performed experiment, density analysis, chlorophyll pigment quantification, imaging-PAM analysis, and prepared samples for sequencing. SF annotated the *Symbiodinium* reference transcriptome. SG and WL analyzed the data. SG and WL wrote the paper. All authors reviewed the manuscript.

## Conflict of Interest Statement

The authors declare that the research was conducted in the absence of any commercial or financial relationships that could be construed as a potential conflict of interest.

## Footnotes

1 https://github.com/sylvainforet/libngs

2 http://seqinr.r-forge.r-project.org/

3 http://cran.r-project.org/package=VennDiagram

4 http://www.r-project.org/

5 http://cran.r-project.org/package=pheatmap

## References

[B1] Aihara Y. Takahashi S. Minagawa J. (2016). Heat induction of cyclic electron flow around photosystem I in the symbiotic dinoflagellate *Symbiodinium*. *Plant Physiol.* 171 522–529. 10.1104/pp.15.01886 26951432 PMC4854689

[B2] Ainsworth T. D. Heron S. F. Ortiz J. C. Mumby P. J. Grech A. Ogawa D. (2016). Climate change disables coral bleaching protection on the Great Barrier Reef. *Science* 352 338–342. 10.1126/science.aac7125 27081069

[B3] Anderson G. H. Alvarez N. D. Gilman C. Jeffares D. C. Trainor V. C. Hanson M. R. (2004). Diversification of genes encoding mei2 -like RNA binding proteins in plants. *Plant Mol. Biol.* 54 653–670. 10.1023/B:PLAN.0000040819.33383.b6 15356386

[B4] Aranda M. Li Y. Liew Y. J. Baumgarten S. Simakov O. Wilson M. C. (2016). Genomes of coral dinoflagellate symbionts highlight evolutionary adaptations conducive to a symbiotic lifestyle. *Sci. Rep.* 6:39734 10.1038/srep39734 PMC5177918 28004835

[B5] Bachvaroff T. R. Concepcion G. T. Rogers C. R. Herman E. M. Delwiche C. F. (2004). Dinoflagellate expressed sequence tag data indicate massive transfer of chloroplast genes to the nuclear genome. *Protist* 155 65–78. 10.1078/1434461000165 15144059

[B6] Baker A. C. (2003). Flexibility and specificity in coral-algal symbiosis: diversity, ecology, and biogeography of *Symbiodinium*. *Annu. Rev. Ecol. Evol. Syst.* 34 661–689. 10.1146/annurev.ecolsys.34.011802.132417

[B7] Barbrook A. C. Voolstra C. R. Howe C. J. (2014). The chloroplast genome of a *Symbiodinium* sp. clade C3 isolate. *Protist* 165 1–13. 10.1016/j.protis.2013.09.006 24316380

[B8] Barshis D. J. Ladner J. T. Oliver T. A. Palumbi S. R. (2014). Lineage-specific transcriptional profiles of *Symbiodinium* spp. unaltered by heat stress in a coral host. *Mol. Biol. Evol.* 31 1343–1352. 10.1093/molbev/msu107 24651035

[B9] Bayer T. Aranda M. Sunagawa S. Yum L. K. DeSalvo M. K. Lindquist E. (2012). *Symbiodinium* transcriptomes: genome insights into the dinoflagellate symbionts of reef-building corals. *PLoS ONE* 7:e35269 10.1371/journal.pone.0035269 PMC3329448 22529998

[B10] Berkelmans R. De’ath G. Kininmonth S. Skirving W. J. (2004). A comparison of the 1998 and 2002 coral bleaching events on the Great Barrier Reef: spatial correlation, patterns, and predictions. *Coral Reefs* 23 74–83. 10.1007/s00338-003-0353-y

[B11] Bissett W. P. Patch J. S. Carder K. L. Lee Z. P. (1997). Pigment packaging and Chl a-specific absorption in high-light oceanic waters. *Limnol. Oceanogr.* 42 961–968. 10.4319/lo.1997.42.5.0961

[B12] Blank R. J. (1987). Cell architecture of the dinoflagellate *Symbiodinium* sp. inhabiting the Hawaiian stony coral *Montipora verrucosa*. *Mar. Biol.* 94 143–155. 10.1007/bf00392906

[B13] Boldt L. Yellowlees D. Leggat W. (2012). Hyperdiversity of genes encoding integral light-harvesting proteins in the dinoflagellate *Symbiodinium*. *PLoS ONE* 7:e47456 10.1371/journal.pone.0047456 PMC3480386 23112815

[B14] Bruno J. Siddon C. Witman J. Colin P. Toscano M. (2001). El Niño related coral bleaching in Palau, Western Caroline Islands. *Coral Reefs* 20 127–136. 10.1007/s003380100151

[B15] Butterfield E. R. Howe C. J. Nisbet R. E. R. (2013). An analysis of dinoflagellate metabolism using EST data. *Protist* 164 218–236. 10.1016/j.protis.2012.09.001 23085481

[B16] Buxton L. Takahashi S. Hill R. Ralph P. J. (2012). Variability in the primary site of photosynthetic damage in *Symbiodinium* sp. (Dinophyceae) exposed to thermal stress. *J. Phycol.* 48 117–126. 10.1111/j.1529-8817.2011.01099.x 27009656

[B17] Castillo-Medina R. E. Islas-Flores T. Thomé P. E. Iglesias-Prieto R. Lin S. Zhang H. (2013). The PsbO homolog from *Symbiodinium kawagutii* (Dinophyceae) characterized using biochemical and molecular methods. *Photosynth. Res.* 115 167–178. 10.1007/s11120-013-9856-8 23708979

[B18] Charif D. Lobry J. R. (2007). “SeqinR 1.0-2: a contributed package to the R project for statistical computing devoted to biological sequences retrieval and analysis,” in *Structural Approaches to Sequence Evolution: Molecules, Networks, Populations* eds Bastolla U. Porto M. Roman H. E. Vendruscolo M. (Berlin: Springer) 207–232.

[B19] Chen H. Boutros P. C. (2011). VennDiagram: a package for the generation of highly-customizable Venn and Euler diagrams in R. *BMC Bioinformatics* 12:35 10.1186/1471-2105-12-35 PMC3041657 21269502

[B20] Chi J. Parrow M. W. Dunthorn M. (2014). Cryptic sex in *Symbiodinium* (Alveolata, Dinoflagellata) is supported by an inventory of meiotic genes. *J. Eukaryot. Microbiol.* 61 322–327. 10.1111/jeu.12110 24904932

[B21] Coffroth M. A. Santos S. R. (2005). Genetic diversity of symbiotic dinoflagellates in the genus *Symbiodinium*. *Protist* 156 19–34. 10.1016/j.protis.2005.02.004 16048130

[B22] Conesa A. Götz S. (2008). Blast2GO: a comprehensive suite for functional analysis in plant genomics. *Int. J. Plant Genomics* 2008:619832 10.1155/2008/619832 PMC2375974 18483572

[B23] Conesa A. Götz S. García-Gómez J. M. Terol J. Talón M. Robles M. (2005). Blast2GO: a universal tool for annotation, visualization and analysis in functional genomics research. *Bioinformatics* 21 3674–3676. 10.1093/bioinformatics/bti610 16081474

[B24] Conesa A. Madrigal P. Tarazona S. Gomez-Cabrero D. Cervera A. McPherson A. (2016). A survey of best practices for RNA-seq data analysis. *Genome Biol.* 17:13 10.1186/s13059-016-0881-8 PMC4728800 26813401

[B25] Cornah J. E. Smith S. M. (2002). “Synthesis and function of glyoxylate cycle enzymes,” in *Plant Peroxisomes: Biochemistry, Cell Biology and Biotechnological Applications* eds Baker A. Graham I. A. (Dordrecht: Springer) 57–101.

[B26] Dankwa D. A. Davis M. J. Kappe S. H. I. Vaughan A. M. (2016). A Plasmodium Mei2-like RNA binding protein is essential for completion of liver stage schizogony. *Infect. Immun.* 84 1336–1345. 10.1128/iai.01417-15 26883588 PMC4862717

[B27] Davy S. K. Allemand D. Weis V. M. (2012). Cell biology of cnidarian-dinoflagellate symbiosis. *Microbiol. Mol. Biol. Rev.* 76 229–261. 10.1128/mmbr.05014-11 22688813 PMC3372257

[B28] Delwiche C. F. (1999). Tracing the thread of plastid diversity through the tapestry of life. *Am. Nat.* 154 S164–S177. 10.1086/303291 10527925

[B29] DeSalvo M. K. Sunagawa S. Voolstra C. R. Medina M. (2010). Transcriptomic responses to heat stress and bleaching in the elkhorn coral *Acropora palmata*. *Mar. Ecol. Prog. Ser.* 402 97–113. 10.3354/meps08372

[B30] Díaz-Almeyda E. Thomé P. E. El Hafidi M. Iglesias-Prieto R. (2011). Differential stability of photosynthetic membranes and fatty acid composition at elevated temperature in *Symbiodinium*. *Coral Reefs* 30 217–225. 10.1007/s00338-010-0691-5

[B31] Engelken J. Brinkmann H. Adamska I. (2010). Taxonomic distribution and origins of the extended LHC (light-harvesting complex) antenna protein superfamily. *BMC Evol. Biol.* 10:233 10.1186/1471-2148-10-233 PMC3020630 20673336

[B32] Fitt W. K. Trench R. K. (1983). The relation of diel patterns of cell division to diel patterns of motility in the symbiotic dinoflagellate *Symbiodinium microadriaticum* freudenthal in culture. *New Phytol.* 94 421–432. 10.1111/j.1469-8137.1983.tb03456.x

[B33] Fry A. M. O’Regan L. Sabir S. R. Bayliss R. (2012). Cell cycle regulation by the NEK family of protein kinases. *J. Cell Sci.* 125(Pt 19) 4423–4433. 10.1242/jcs.111195 23132929 PMC3500863

[B34] Fu L. Niu B. Zhu Z. Wu S. Li W. (2012). CD-HIT: accelerated for clustering the next-generation sequencing data. *Bioinformatics* 28 3150–3152. 10.1093/bioinformatics/bts565 23060610 PMC3516142

[B35] Fujise L. Yamashita H. Suzuki G. Sasaki K. Liao L. M. Koike K. (2014). Moderate thermal stress causes active and immediate expulsion of photosynthetically damaged zooxanthellae (*Symbiodinium*) from corals. *PLoS ONE* 9:e114321 10.1371/journal.pone.0114321 PMC4262390 25493938

[B36] Gibellini F. Smith T. K. (2010). The Kennedy pathway—*De novo* synthesis of phosphatidylethanolamine and phosphatidylcholine. *IUBMB Life* 62 414–428. 10.1002/iub.337 20503434

[B37] Gierz S. L. Gordon B. R. Leggat W. (2016). Integral light-harvesting complex expression in *Symbiodinium* within the coral *Acropora aspera* under thermal stress. *Sci. Rep.* 6:25081 10.1038/srep25081 PMC4846871 27117333

[B38] Grabherr M. G. Haas B. J. Yassour M. Levin J. Z. Thompson D. A. Amit I. (2011). Full-length transcriptome assembly from RNA-Seq data without a reference genome. *Nat. Biotechnol.* 29 644–652. 10.1038/nbt.1883 21572440 PMC3571712

[B39] Haas B. J. Papanicolaou A. Yassour M. Grabherr M. Blood P. D. Bowden J. (2013). *De novo* transcript sequence reconstruction from RNA-Seq: reference generation and analysis with Trinity. *Nat. Protoc.* 8 1494–1512. 10.1038/nprot.2013.084 23845962 PMC3875132

[B40] Hackett J. D. Anderson D. M. Erdner D. L. Bhattacharya D. (2004). Dinoflagellates: a remarkable evolutionary experiment. *Am. J. Bot.* 91 1523–1534. 10.3732/ajb.91.10.1523 21652307

[B41] Hill R. Takahashi S. (2014). Photosystem II recovery in the presence and absence of chloroplast protein repair in the symbionts of corals exposed to bleaching conditions. *Coral Reefs* 33 1101–1111. 10.1007/s00338-014-1188-4

[B42] Hisabori T. Motohashi K. Hosoya-Matsuda N. Ueoka-Nakanishi H. Romano P. G. N. (2007). Towards a functional dissection of thioredoxin networks in plant cells. *Photochem. Photobiol.* 83 145–151. 10.1562/2006-02-27-IR-816 16706599

[B43] Hoegh-Guldberg O. (1999). Climate change, coral bleaching and the future of the world’s coral reefs. *Mar. Freshw. Res.* 50 839–866. 10.1071/MF99078

[B44] Ifuku K. Ido K. Sato F. (2011). Molecular functions of PsbP and PsbQ proteins in the photosystem II supercomplex. *J. Photochem. Photobiol. B Biol.* 104 158–164. 10.1016/j.jphotobiol.2011.02.006 21376623

[B45] Ifuku K. Noguchi T. (2016). Structural coupling of extrinsic proteins with the oxygen-evolving center in photosystem II. *Front. Plant Sci.* 7:84 10.3389/fpls.2016.00084 PMC4743485 26904056

[B46] Iglesias-Prieto R. Govind N. S. Trench R. K. (1993). Isolation and characterization of three membrane-bound chlorophyll-protein complexes from four dinoflagellate species. *Philos. Trans. R. Soc. B Biol. Sci.* 340 381–392. 10.1098/rstb.1993.0080

[B47] Iglesias-Prieto R. Matta J. L. Robins W. A. Trench R. K. (1992). Photosynthetic response to elevated temperature in the symbiotic dinoflagellate *Symbiodinium microadriaticum* in culture. *Proc. Natl. Acad. Sci. U.S.A.* 89 10302–10305. 10.1073/pnas.89.21.10302 11607337 PMC50326

[B48] Iglesias-Prieto R. Trench R. K. (1997). Acclimation and adaptation to irradiance in symbiotic dinoflagellates. II. Response of chlorophyll–protein complexes to different photon-flux densities. *Mar. Biol.* 130 23–33. 10.1007/s002270050221

[B49] Iida S. Kobiyama A. Ogata T. Murakami A. (2008). The D1 and D2 proteins of dinoflagellates: unusually accumulated mutations which influence on PSII photoreaction. *Photosynth. Res.* 98 415–425. 10.1007/s11120-008-9378-y 18855112

[B50] Jeffares D. C. Phillips M. J. Moore S. Veit B. (2004). A description of the Mei2-like protein family; structure, phylogenetic distribution and biological context. *Dev. Genes Evol.* 214 149–158. 10.1007/s00427-004-0384-6 14986133

[B51] Jeffrey S. W. Humphrey G. F. (1975). New spectrophotometric equations for determining chlorophylls a, b, c1, and c2 in higher plants, algae and natural phytoplankton. *Biochem. Physiol. Pfl.* 167 191–194.

[B52] Karim W. Nakaema S. Hidaka M. (2015). Temperature effects on the growth rates and photosynthetic activities of *Symbiodinium* cells. *J. Mar. Sci. Eng.* 3:368 10.3390/jmse3020368

[B53] Kaur J. Sebastian J. Siddiqi I. (2006). The *Arabidopsis*-mei2-like genes play a role in meiosis and vegetative growth in *Arabidopsis*. *Plant Cell* 18 545–559. 10.1105/tpc.105.039156 16473967 PMC1383632

[B54] Kenkel C. D. Meyer E. Matz M. V. (2013). Gene expression under chronic heat stress in populations of the mustard hill coral (*Porites astreoides*) from different thermal environments. *Mol. Ecol.* 22 4322–4334. 10.1111/mec.12390 23899402

[B55] Kneeland J. Hughen K. Cervino J. Hauff B. Eglinton T. (2013). Lipid biomarkers in *Symbiodinium* dinoflagellates: new indicators of thermal stress. *Coral Reefs* 32 923–934. 10.1007/s00338-013-1076-3

[B56] Kolde R. (2015). *pheatmap: Pretty Heatmaps. R Package Version* 1.0.8.

[B57] Kornberg H. L. Madsen N. B. (1958). The metabolism of C(2) compounds in micro-organisms. 3. Synthesis of malate from acetate via the glyoxylate cycle. *Biochem. J.* 68 549–557. 10.1042/bj0680549 13522658 PMC1200390

[B58] Krueger T. Becker S. Pontasch S. Dove S. Hoegh-Guldberg O. Leggat W. (2014). Antioxidant plasticity and thermal sensitivity in four types of *Symbiodinium* sp. *J. Phycol.* 50 1035–1047. 10.1111/jpy.12232 26988785

[B59] Krueger T. Fisher P. L. Becker S. Pontasch S. Dove S. Hoegh-Guldberg O. (2015). Transcriptomic characterization of the enzymatic antioxidants FeSOD, MnSOD, APX and KatG in the dinoflagellate genus *Symbiodinium*. *BMC Evol. Biol.* 15:1–20. 10.1186/s12862-015-0326-0 25887897 PMC4416395

[B60] Kültz D. (2005). Molecular and evolutionary basis of the cellular stress response. *Annu. Rev. Physiol.* 67 225–257. 10.1146/annurev.physiol.67.040403.103635 15709958

[B61] Ladner J. Barshis D. Palumbi S. (2012). Protein evolution in two co-occurring types of *Symbiodinium*: an exploration into the genetic basis of thermal tolerance in *Symbiodinium* clade D. *BMC Evol. Biol.* 12:217 10.1186/1471-2148-12-217 PMC3740780 23145489

[B62] Leggat W. Hoegh-Guldberg O. Dove S. Yellowlees D. (2007). Analysis of an EST library from the dinoflagellate (*Symbiodinium* sp.) symbiont of reef-building corals. *J. Phycol.* 43 1010–1021. 10.1111/j.1529-8817.2007.00387.x

[B63] Leggat W. Seneca F. Wasmund K. Ukani L. Yellowlees D. Ainsworth T. D. (2011a). Differential responses of the coral host and their algal symbiont to thermal stress. *PLoS ONE* 6:e26687 10.1371/journal.pone.0026687 PMC3200360 22039532

[B64] Leggat W. Yellowlees D. Medina M. (2011b). Recent progress in *Symbiodinium* transcriptomics. *J. Exp. Mar. Biol. Ecol.* 408 120–125. 10.1016/j.jembe.2011.07.032

[B65] Lesser M. P. (2006). Oxidative stress in marine environments: biochemistry and physiological ecology. *Annu. Rev. Physiol.* 68 253–278. 10.1146/annurev.physiol.68.040104.110001 16460273

[B66] Levin R. A. Beltran V. H. Hill R. Kjelleberg S. McDougald D. Steinberg P. D. (2016). Sex, scavengers, and chaperones: transcriptome secrets of divergent *Symbiodinium* thermal tolerances. *Mol. Biol. Evol.* 33:3032 10.1093/molbev/msw119 PMC7297275 27738273

[B67] Li P. Piao Y. Shon H. S. Ryu K. H. (2015). Comparing the normalization methods for the differential analysis of Illumina high-throughput RNA-Seq data. *BMC Bioinformatics* 16:347 10.1186/s12859-015-0778-7 PMC4625728 26511205

[B68] Lin S. Cheng S. Song B. Zhong X. Lin X. Li W. (2015). The *Symbiodinium kawagutii* genome illuminates dinoflagellate gene expression and coral symbiosis. *Science* 350 691–694. 10.1126/science.aad0408 26542574

[B69] Maruyama S. Shoguchi E. Satoh N. Minagawa J. (2015). Diversification of the light-harvesting complex gene family via intra- and intergenic duplications in the coral symbiotic alga *Symbiodinium*. *PLoS ONE* 10:e0119406 10.1371/journal.pone.0119406 PMC4351107 25741697

[B70] McBride B. B. Muller-Parker G. Jakobsen H. H. (2009). Low thermal limit of growth rate of *Symbiodinium californium* (dinophyta) in culture may restrict the symbiont to southern populations of its host anemones (*Anthopleura* spp.; *anthozoa, cnidaria)*. *J. Phycol.* 45 855–863. 10.1111/j.1529-8817.2009.00716.x 27034215

[B71] McGinley M. P. Aschaffenburg M. D. Pettay D. T. Smith R. T. LaJeunesse T. C. Warner M. E. (2012). Transcriptional response of two core photosystem genes in *Symbiodinium* spp. exposed to thermal stress. *PLoS ONE* 7:e50439 10.1371/journal.pone.0050439 PMC3517614 23236373

[B72] Meyer E. Aglyamova G. V. Wang S. Buchanan-Carter J. Abrego D. Colbourne J. K. (2009). Sequencing and de novo analysis of a coral larval transcriptome using 454 GSFlx. *BMC Genomics* 10:219 10.1186/1471-2164-10-219 PMC2689275 19435504

[B73] Mohamed A. R. Cumbo V. Harii S. Shinzato C. Chan C. X. Ragan M. A. (2016). The transcriptomic response of the coral *Acropora digitifera* to a competent *Symbiodinium* strain: the symbiosome as an arrested early phagosome. *Mol. Ecol.* 25 3127–3141. 10.1111/mec.13659 27094992

[B74] Mortazavi A. Williams B. A. McCue K. Schaeffer L. Wold B. (2008). Mapping and quantifying mammalian transcriptomes by RNA-Seq. *Nat. Methods* 5 621–628. 10.1038/nmeth.1226 18516045 PMC13303166

[B75] Mungpakdee S. Shinzato C. Takeuchi T. Kawashima T. Koyanagi R. Hisata K. (2014). Massive gene transfer and extensive RNA editing of a symbiotic dinoflagellate plastid genome. *Genome Biol. Evol.* 6 1408–1422. 10.1093/gbe/evu109 24881086 PMC4079212

[B76] Murata N. Takahashi S. Nishiyama Y. Allakhverdiev S. I. (2007). Photoinhibition of photosystem II under environmental stress. *Biochim. Biophys. Acta* 1767 414–421. 10.1016/j.bbabio.2006.11.019 17207454

[B77] Nigg E. A. (2001). Mitotic kinases as regulators of cell division and its checkpoints. *Nat. Rev. Mol. Cell Biol.* 2 21–32. 10.1038/35048096 11413462

[B78] Nishiyama Y. Allakhverdiev S. I. Murata N. (2011). Protein synthesis is the primary target of reactive oxygen species in the photoinhibition of photosystem II. *Physiol. Plant.* 142 35–46. 10.1111/j.1399-3054.2011.01457.x 21320129

[B79] Ogawa D. Bobeszko T. Ainsworth T. Leggat W. (2013). The combined effects of temperature and CO2 lead to altered gene expression in *Acropora aspera*. *Coral Reefs* 32 895–907. 10.1007/s00338-013-1046-9

[B80] Parkinson J. E. Baumgarten S. Michell C. T. Baums I. B. LaJeunesse T. C. Voolstra C. R. (2016). Gene expression variation resolves species and individual strains among coral-associated dinoflagellates within the genus *Symbiodinium*. *Genome Biol. Evol.* 8 665–680. 10.1093/gbe/evw019 26868597 PMC4824173

[B81] Pilkis S. J. Granner D. K. (1992). Molecular physiology of the regulation of hepatic gluconeogenesis and glycolysis. *Annu. Rev. Physiol.* 54 885–909. 10.1146/annurev.ph.54.030192.004321 1562196

[B82] Pochon X. Gates R. D. (2010). A new *Symbiodinium* clade (Dinophyceae) from soritid foraminifera in Hawai’i. *Mol. Phylogenet. Evol.* 56 492–497. 10.1016/j.ympev.2010.03.040 20371383

[B83] Pochon X. Stat M. Takabayashi M. Chasqui L. Chauka L. J. Logan D. D. K. (2010). Comparison of endosymbiotic and free-living *Symbiodinium* (Dinophyceae) diversity in a Hawaiian reef environment. *J. Phycol.* 46 53–65. 10.1111/j.1529-8817.2009.00797.x

[B84] Poirier Y. Antonenkov V. D. Glumoff T. Hiltunen J. K. (2006). Peroxisomal β-oxidation—A metabolic pathway with multiple functions. *Biochim. Biophys. Acta* 1763 1413–1426. 10.1016/j.bbamcr.2006.08.034 17028011

[B85] R Core Team (2014). *R: A Language and Environment for Statistical Computing*. Vienna: R Foundation for Statistical Computing.

[B86] Ralph P. J. Gademann R. Larkum A. W. D. (2001). Zooxanthellae expelled from bleached corals at 33°C are photosynthetically competent. *Mar. Ecol. Prog. Ser.* 220 163–168. 10.3354/meps220163

[B87] Ralph P. J. Larkum A. W. D. Kühl M. (2005). Temporal patterns in effective quantum yield of individual zooxanthellae expelled during bleaching. *J. Exp. Mar. Biol. Ecol.* 316 17–28. 10.1016/j.jembe.2004.10.003

[B88] Rochaix J.-D. (2011). Assembly of the photosynthetic apparatus. *Plant Physiol.* 155 1493–1500. 10.1104/pp.110.169839 21239619 PMC3091127

[B89] Rosic N. N. Ling E. Y. Chan C. K. Lee H. C. Kaniewska P. Edwards D. (2014). Unfolding the secrets of coral-algal symbiosis. *ISME J.* 9 844–856. 10.1038/ismej.2014.182 PMC4817714 25343511

[B90] Rosic N. N. Pernice M. Dove S. Dunn S. Hoegh-Guldberg O. (2011). Gene expression profiles of cytosolic heat shock proteins Hsp70 and Hsp90 from symbiotic dinoflagellates in response to thermal stress: possible implications for coral bleaching. *Cell Stress Chaperones* 16 69–80. 10.1007/s12192-010-0222-x 20821176 PMC3024090

[B91] Sammarco P. W. Strychar K. B. (2013). Responses to high seawater temperatures in zooxanthellate octocorals. *PLoS ONE* 8:e54989 10.1371/journal.pone.0054989 PMC3566138 23405104

[B92] Santos S. R. Coffroth M. A. (2003). Molecular genetic evidence that dinoflagellates belonging to the genus *Symbiodinium* freudenthal are haploid. *Biol. Bull.* 204 10–20. 10.2307/1543491 12588740

[B93] Shi W. Bogdanov M. Dowhan W. Zusman D. R. (1993). The pss and psd genes are required for motility and chemotaxis in *Escherichia coli*. *J. Bacteriol.* 175 7711–7714. 10.1128/jb.175.23.7711-7714.1993 8244943 PMC206932

[B94] Shinzato C. Mungpakdee S. Satoh N. Shoguchi E. (2014). A genomic approach to coral-dinoflagellate symbiosis: studies of *Acropora digitifera* and *Symbiodinium minutum*. *Front. Microbiol.* 5:336 10.3389/fmicb.2014.00336 PMC4083563 25071748

[B95] Shoguchi E. Shinzato C. Hisata K. Satoh N. Mungpakdee S. (2015). The large mitochondrial genome of *Symbiodinium minutum* reveals conserved noncoding sequences between dinoflagellates and apicomplexans. *Genome Biol. Evol.* 7 2237–2244. 10.1093/gbe/evv137 26199191 PMC4558855

[B96] Shoguchi E. Shinzato C. Kawashima T. Gyoja F. Mungpakdee S. Koyanagi R. (2013). Draft assembly of the *Symbiodinium minutum* nuclear genome reveals dinoflagellate gene structure. *Curr. Biol.* 23 1399–1408. 10.1016/j.cub.2013.05.062 23850284

[B97] Solomon S. Qin D. Manning M. Chen Z. Marquis M. Averyt K. B. (2007). *Contribution of Working Group I to the Fourth Assessment Report of the Intergovernmental Panel on Climate Change, 2007*. Cambridge: Cambridge University Press.

[B98] Stat M. Morris E. Gates R. D. (2008). Functional diversity in coral–dinoflagellate symbiosis. *Proc. Natl. Acad. Sci. U.S.A.* 105 9256–9261. 10.1073/pnas.0801328105 18591663 PMC2453720

[B99] Sutton D. C. Hoegh-Guldberg O. (1990). Host-zooxanthella interactions in four temperate marine invertebrate symbioses: assessment of effect of host extracts on symbionts. *Biol. Bull.* 178 175–186. 10.2307/1541975 29314935

[B100] Takahashi S. Whitney S. Itoh S. Maruyama T. Badger M. (2008). Heat stress causes inhibition of the de novo synthesis of antenna proteins and photobleaching in cultured *Symbiodinium*. *Proc. Natl. Acad. Sci. U.S.A.* 105 4203–4208. 10.1073/pnas.0708554105 18322010 PMC2393757

[B101] Takahashi S. Yoshioka-Nishimura M. Nanba D. Badger M. R. (2013). Thermal acclimation of the symbiotic alga *Symbiodinium* spp. alleviates photobleaching under heat stress. *Plant Physiol.* 161 477–485. 10.1104/pp.112.207480 23170037 PMC3532276

[B102] Tchernov D. Gorbunov M. Y. de Vargas C. Narayan Yadav S. Milligan A. J. Haggblom M. (2004). Membrane lipids of symbiotic algae are diagnostic of sensitivity to thermal bleaching in corals. *Proc. Natl. Acad. Sci. U.S.A.* 101 13531–13535. 10.1073/pnas.0402907101 15340154 PMC518791

[B103] Venn A. A. Loram J. E. Douglas A. E. (2008). Photosynthetic symbioses in animals. *J. Exp. Biol.* 59 1069–1080. 10.1093/jxb/erm328 18267943

[B104] Von Wettstein D. Gough S. Kannangara C. G. (1995). Chlorophyll biosynthesis. *Plant Cell* 7 1039–1057. 10.1105/tpc.7.7.1039 12242396 PMC160907

[B105] Voolstra C. R. Schwarz J. A. Schnetzer J. Sunagawa S. Desalvo M. K. Szmant A. M. (2009a). The host transcriptome remains unaltered during the establishment of coral-algal symbioses. *Mol. Ecol.* 18 1823–1833. 10.1111/j.1365-294X.2009.04167.x 19317843

[B106] Voolstra C. R. Sunagawa S. Schwarz J. A. Coffroth M. A. Yellowlees D. Leggat W. (2009b). Evolutionary analysis of orthologous cDNA sequences from cultured and symbiotic dinoflagellate symbionts of reef-building corals (Dinophyceae: *Symbiodinium*). *Comp. Biochem. Physiol. Part D Genomics Proteomics* 4 67–74. 10.1016/j.cbd.2008.11.001 20403741

[B107] Wang L.-H. Lee H.-H. Fang L.-S. Mayfield A. B. Chen C.-S. (2013). Fatty acid and phospholipid syntheses are prerequisites for the cell cycle of *Symbiodinium* and their endosymbiosis within sea anemones. *PLoS ONE* 8:e72486 10.1371/journal.pone.0072486 PMC3756969 24009685

[B108] Warner M. E. Fitt W. K. Schmidt G. W. (1999). Damage to photosystem II in symbiotic dinoflagellates: a determinant of coral bleaching. *Proc. Natl. Acad. Sci. U.S.A.* 96 8007–8012. 10.1073/pnas.96.14.8007 10393938 PMC22178

[B109] Wilkinson S. P. Fisher P. L. van Oppen M. J. Davy S. K. (2015). Intra-genomic variation in symbiotic dinoflagellates: recent divergence or recombination between lineages? *BMC Evol. Biol.* 15:46 10.1186/s12862-015-0325-1 PMC4381663 25887753

[B110] Xiang T. Nelson W. Rodriguez J. Tolleter D. Grossman A. R. (2015). *Symbiodinium* transcriptome and global responses of cells to immediate changes in light intensity when grown under autotrophic or mixotrophic conditions. *Plant J.* 82 67–80. 10.1111/tpj.12789 25664570

[B111] Yellowlees D. Rees T. A. V. Leggat W. (2008). Metabolic interactions between algal symbionts and invertebrate hosts. *Plant Cell Environ.* 31 679–694. 10.1111/j.1365-3040.2008.01802.x 18315536

