# Supplementary material for: Transcriptomic Analysis of Thermally Stressed Symbiodinium Reveals Differential Expression of Stress and Metabolism Genes
Source: Front Plant Sci. 2017 Feb 28;8:271. doi: 10.3389/fpls.2017.00271 (PMC5328969; doi:10.3389/fpls.2017.00271)
Supplement: Supplementary file 1 [file Table_1.DOCX]

Supplementary Material

Transcriptomic analysis of thermally stressed *Symbiodinium* reveals differential expression of stress and metabolism genes

Sarah L. Gierz^1, 2,^ *, Sylvain Forêt^3, 4^ and William Leggat^1, 2, 3^

* Correspondence: Sarah L. Gierz: sarah.gierz@my.jcu.edu.au

**Supplementary Table 1.** Summary of *Symbiodinium* sequencing projects.

| Species (strain) | Clade/ Type | Host/ Culture | Dataset/ Conditions | No. contigs/ Gene content | Sequencing technology | Citation |
| --- | --- | --- | --- | --- | --- | --- |
| Genomes | | | | | | |
| *Symbiodinium minutum* (Mf1.05b.01) | B1 | Culture | Nuclear genome | 41,925 | 454, Illumina | Shoguchi *et al.* (2013) |
| *Symbiodinium* sp. | C3 | *Agaricia sp*. | Plastid genome | 13 minicircles | Sanger | Barbrook *et al.* (2014) |
| *Symbiodinium minutum* (Mf1.05b.01) | B1 | Culture | Plastid genome | 109  (14 minicircles) | Sanger, Illumina | Mungpakdee *et al.* (2014) |
| *Symbiodinium minutum* (Mf1.05b.01) | B1 | Culture | Mitochondrial genome |  | 454, Illumina | Shoguchi *et al.* (2015) |
| *Symbiodinium kawagutii* | F1 | Culture | Nuclear genome | 36,850  70,985 ESTs | Sanger, 454 | Lin *et al.* (2015) |
| *Symbiodinium microadriaticum* (CCMP2467) | A1 | Culture | Nuclear genome | 49,109 | Illumina | Aranda *et al.* (2016) |
| Transcriptomes | | | | | | |
| *Symbiodinium* sp. | C3 | *Acropora aspera* | Varied stress conditions  EST library | 5184 | Sanger | Leggat *et al.* (2007) |
| *Symbiodinium* sp. (CassKB8) | A1 | Culture | EST library | 2653 | Sanger | Voolstra *et al.* (2009) |
| *Symbiodinium* sp. | A3 | *Aiptasia pallida* | EST library | 127 | Sanger | Sunagawa *et al.* (2009) |
| *Symbiodinium* sp. (CassKB8) | A1 | Culture | Reference transcriptomes | 57,676 | 454 | Bayer *et al.* (2012) |
| *Symbiodinium* sp. (Mf1.05b) | B1 | Culture |  | 56,198 |  |  |
| *Symbiodinium* spp. | C | *Acropora hyacinthus* | Reference transcriptome | 26,986 | Illumina | Ladner *et al.* (2012) |
| *Symbiodinium* spp. | D |  |  | 23,777 |  |  |
| *Symbiodinium microadriaticum* (CCMP2467) | A1 | Culture | Varied stressors (< 24 h)  smRNAs (miRNAs and siRNAs) | 58,694 | Illumina | Baumgarten *et al.* (2013) |
| *Symbiodinium kawagutii* | F1 | Culture | 5’-cap selective full length cDNA library | 7,536 | Sanger | Zhang *et al.* (2013) |
| *Symbiodinium* sp. | C3K | *Acropora hyacinthus* | DEG  Thermal stress (+2.7 °C (mean 31.9 °C), 3 d)  Reciprocal transplant | DEG identified using Ladner *et al.* (2012) as reference | Illumina | Barshis *et al.* (2014) |
| *Symbiodinium* sp. | D2 |  |  |  |  |  |
| *Symbiodinium* sp. | C3 | *Acropora aspera* | DEG  Thermal stress (+6 °C (30 °C), 1 & 3 d)  Nutrient stress (ammonium + 20 μM) | DEG identified using DiffKAP method | Illumina | Rosic *et al.* (2014a) |
| *Symbiodinium* sp. | A2 | Culture | Reference transcriptomes | 29,846 | Illumina | Rosic *et al.* (2014b) |
| *Symbiodinium* sp. | B2 |  |  | 42,233 |  |  |
| *Symbiodinium* sp. | C1 |  |  | 46,892 |  |  |
| *Symbiodinium* sp. | D1 |  |  | 42,885 |  |  |
| *Symbiodinium* sp. | C15 | *Porites australiensis* | Reference transcriptome | 26,627 | Illumina | Shinzato *et al.* (2014a) |
| *Symbiodinium* spp. (SSB01) | B | Culture | Light stress (0 - 500 μmol photons m^-2^ s^-1^)  +/ - Nutrient supplement | 59,669 | Illumina | Xiang *et al.* (2015) |
| *Symbiodinium aenigmaticum*  (mac04-487) | B19 | Culture | DEG  Species specific comparison of orthologous genes | 45,343 | Illumina | Parkinson *et al.* (2016) |
| *Symbiodinium minutum*  (mac703, Mf1.05b, rt002 and rt351) | B1 |  |  | 51,199 |  |  |
| *Symbiodinium pseudominutum* (rt146) | B1 |  |  | 47,411 |  |  |
| *Symbiodinium psygmophilum*  (HIAp, Mf10.14b.02, PurPFlex, rt141) | B19 |  |  | 50,745 |  |  |
| *Symbiodinium* sp. | C1 | Culture | Thermal stress  (+5 °C (32 °C), 9 & 13 d) | 106,097 | Illumina | Levin *et al.* (2016) |
| *Symbiodinium* sp. | C1 |  |  | 93,377 |  |  |
| *Symbiodinium* sp. |  | *Porites astreoides* | Host developmental stages | 145,570 | Illumina | Mansour *et al.* (2016) |

Table adapted from Shinzato *et al.* (2014b) and updated with current datasets available.

References:

Aranda, M, Li, Y, Liew, YJ, Baumgarten, S, Simakov, O, Wilson, MC, et al. (2016). Genomes of coral dinoflagellate symbionts highlight evolutionary adaptations conducive to a symbiotic lifestyle. *Scientific Reports* 6**,** 39734. doi: 10.1038/srep39734.

Barbrook, AC, Voolstra, CR, and Howe, CJ (2014). The chloroplast genome of a *Symbiodinium* sp. clade C3 isolate. *Protist* 165(1)**,** 1-13. doi: 10.1016/j.protis.2013.09.006.

Barshis, DJ, Ladner, JT, Oliver, TA, and Palumbi, SR (2014). Lineage-specific transcriptional profiles of *Symbiodinium* spp. unaltered by heat stress in a coral host. *Molecular Biology and Evolution* 31(6)**,** 1343-1352. doi: 10.1093/molbev/msu107.

Baumgarten, S, Bayer, T, Aranda, M, Liew, Y, Carr, A, Micklem, G, et al. (2013). Integrating microRNA and mRNA expression profiling in *Symbiodinium microadriaticum*, a dinoflagellate symbiont of reef-building corals. *BMC Genomics* 14(1)**,** 704. doi: 10.1186/1471-2164-14-704.

Bayer, T, Aranda, M, Sunagawa, S, Yum, LK, DeSalvo, MK, Lindquist, E, et al. (2012). *Symbiodinium* transcriptomes: Genome insights into the dinoflagellate symbionts of reef-building corals. *PLoS ONE* 7(4)**,** e35269. doi: 10.1371/journal.pone.0035269.

Ladner, J, Barshis, D, and Palumbi, S (2012). Protein evolution in two co-occurring types of *Symbiodinium*: an exploration into the genetic basis of thermal tolerance in *Symbiodinium* clade D. *BMC Evolutionary Biology* 12(1)**,** 217. doi: 10.1186/1471-2148-12-217.

Leggat, W, Hoegh-Guldberg, O, Dove, S, and Yellowlees, D (2007). Analysis of an EST library from the dinoflagellate (*Symbiodinium* sp.) symbiont of reef-building corals. *Journal of Phycology* 43(5)**,** 1010-1021. doi: 10.1111/j.1529-8817.2007.00387.x.

Levin, RA, Beltran, VH, Hill, R, Kjelleberg, S, McDougald, D, Steinberg, PD, et al. (2016). Sex, scavengers, and chaperones: Transcriptome secrets of divergent *Symbiodinium* thermal tolerances. *Molecular Biology and Evolution* 33(11)**,** 3032. doi: 10.1093/molbev/msw119.

Lin, S, Cheng, S, Song, B, Zhong, X, Lin, X, Li, W, et al. (2015). The *Symbiodinium kawagutii* genome illuminates dinoflagellate gene expression and coral symbiosis. *Science* 350(6261)**,** 691-694. doi: 10.1126/science.aad0408.

Mansour, TA, Rosenthal, JJC, Brown, CT, and Roberson, LM (2016). Transcriptome of the Caribbean stony coral *Porites astreoides* from three developmental stages. *GigaScience* 5(1)**,** 33. doi: 10.1186/s13742-016-0138-1.

Mungpakdee, S, Shinzato, C, Takeuchi, T, Kawashima, T, Koyanagi, R, Hisata, K, et al. (2014). Massive gene transfer and extensive RNA editing of a symbiotic dinoflagellate plastid genome. *Genome Biology and Evolution* 6(6)**,** 1408-1422. doi: 10.1093/gbe/evu109.

Parkinson, JE, Baumgarten, S, Michell, CT, Baums, IB, LaJeunesse, TC, and Voolstra, CR (2016). Gene expression variation resolves species and individual strains among coral-associated dinoflagellates within the genus *Symbiodinium*. *Genome Biology and Evolution* 8**,** 665-680. doi: 10.1093/gbe/evw019.

Rosic, NN, Kaniewska, P, Chan, C, Ling, EY, Edwards, D, Dove, S, et al. (2014a). Early transcriptional changes in the reef-building coral *Acropora aspera* in response to thermal and nutrient stress. *BMC Genomics* 15(1)**,** 1052. doi: 10.1186/1471-2164-15-1052.

Rosic, NN, Ling, EY, Chan, CK, Lee, HC, Kaniewska, P, Edwards, D, et al. (2014b). Unfolding the secrets of coral-algal symbiosis. *Isme j* 9**,** 844-856. doi: 10.1038/ismej.2014.182.

Shinzato, C, Inoue, M, and Kusakabe, M (2014a). A snapshot of a coral “holobiont”: A transcriptome assembly of the scleractinian coral, *Porites*, captures a wide variety of genes from both the host and symbiotic zooxanthellae. *PLoS ONE* 9(1)**,** e85182. doi: 10.1371/journal.pone.0085182.

Shinzato, C, Mungpakdee, S, Satoh, N, and Shoguchi, E (2014b). A genomic approach to coral-dinoflagellate symbiosis: studies of *Acropora digitifera* and *Symbiodinium minutum*. *Frontiers in Microbiology* 5(336). doi: 10.3389/fmicb.2014.00336.

Shoguchi, E, Shinzato, C, Hisata, K, Satoh, N, and Mungpakdee, S (2015). The large mitochondrial genome of *Symbiodinium minutum* reveals conserved noncoding sequences between dinoflagellates and apicomplexans. *Genome Biology and Evolution* 7(8)**,** 2237-2244. doi: 10.1093/gbe/evv137.

Shoguchi, E, Shinzato, C, Kawashima, T, Gyoja, F, Mungpakdee, S, Koyanagi, R, et al. (2013). Draft assembly of the *Symbiodinium minutum* nuclear genome reveals dinoflagellate gene structure. *Current Biology* 23(15)**,** 1399-1408. doi: 10.1016/j.cub.2013.05.062.

Sunagawa, S, Wilson, E, Thaler, M, Smith, M, Caruso, C, Pringle, J, et al. (2009). Generation and analysis of transcriptomic resources for a model system on the rise: the sea anemone *Aiptasia pallida* and its dinoflagellate endosymbiont. *BMC Genomics* 10(1)**,** 258. doi: DOI: 10.1186/1471-2164-10-258.

Voolstra, CR, Sunagawa, S, Schwarz, JA, Coffroth, MA, Yellowlees, D, Leggat, W, et al. (2009). Evolutionary analysis of orthologous cDNA sequences from cultured and symbiotic dinoflagellate symbionts of reef-building corals (Dinophyceae: *Symbiodinium*). *Comparative Biochemistry and Physiology Part D: Genomics and Proteomics* 4(2)**,** 67-74. doi: 10.1016/j.cbd.2008.11.001.

Xiang, T, Nelson, W, Rodriguez, J, Tolleter, D, and Grossman, AR (2015). *Symbiodinium* transcriptome and global responses of cells to immediate changes in light intensity when grown under autotrophic or mixotrophic conditions. *The Plant Journal* 82(1)**,** 67-80. doi: 10.1111/tpj.12789.

Zhang, H, Zhuang, Y, Gill, J, and Lin, S (2013). Proof that dinoflagellate spliced leader (DinoSL) is a useful hook for fishing dinoflagellate transcripts from mixed microbial samples: *Symbiodinium kawagutii* as a case study. *Protist* 164(4)**,** 510-527. doi: 10.1016/j.protis.2013.04.002.
