# Supplementary material for: Transcriptomic Analysis of Thermally Stressed Symbiodinium Reveals Differential Expression of Stress and Metabolism Genes
Source: Front Plant Sci. 2017 Feb 28;8:271. doi: 10.3389/fpls.2017.00271 (PMC5328969; doi:10.3389/fpls.2017.00271)
Supplement: Supplementary file 6 [file Data_Sheet_1.DOCX]

Supplementary Material

Transcriptomic analysis of thermally stressed *Symbiodinium* reveals differential expression of stress and metabolism genes

Sarah L. Gierz^1, 2,^ *, Sylvain Forêt^3, 4^ and William Leggat^1, 2, 3^

* Correspondence: Sarah L. Gierz: sarah.gierz@my.jcu.edu.au

**Supplementary Figure 1.** Experimental sampling regime.

**Supplementary Figure 2.** Visualization of the distribution of molecular function GO classifications for the 2,798 genes differentially expressed at all time points in *Symbiodinium* exposed to thermal stress (FDR < 0.05). GO annotation graph produced using Blast2GO, GO categories displayed at ontology level 3 and slices smaller than 2% grouped into the ‘other’ term, numbers displayed represent the number of sequences assigned to each ontology category.

**Supplementary Figure 3.** Visualization of the distribution of cellular component GO classifications for the 2,798 genes differentially expressed at all time points in *Symbiodinium* exposed to thermal stress (FDR < 0.05). GO annotation graph produced using Blast2GO, GO categories displayed at ontology level 3 and slices smaller than 2% grouped into the ‘other’ term, numbers displayed represent the number of sequences assigned to each ontology category.

**Supplementary Figure 4.** Distribution of biological process GO terms of differentially expressed transcripts in thermally stressed *Symbiodinium*. Data displayed for 2,798 transcripts that were differentially expressed (FDR < 0.05) at day four, nineteen and twenty-eight. Transcripts that displayed increased expression at all time points (light grey bars), transcripts that displayed decreased expression at all time points (dark grey bars) and transcripts that displayed mixed expression at all time points (black bars) are shown.
